# Supplementary material for: Effect of exercise training on heath, quality of life, exercise capacity in juvenile idiopathic arthritis: a meta-analysis of randomized controlled trials
Source: Pediatr Rheumatol Online J. 2024 Mar 4;22:33. doi: 10.1186/s12969-024-00967-3 (PMC10910763; doi:10.1186/s12969-024-00967-3)
Supplement: Supplementary file 1 — Supplementary Material 1 [file 12969_2024_967_MOESM1_ESM.docx]

**Effect of exercise training on heath, quality of life, exercise capacity in juvenile idiopathic arthritis: a meta-analysis of randomized controlled trials**

**Supplementary**

**appendix**

Catalogue

[1.Search strategy 1](#_Toc135167358)

[1.1 PubMed 1](#_Toc135167359)

[1.2 Embase 4](#_Toc135167360)

[1.3 Web of Science 5](#_Toc135167361)

[1.4 Cochrane library 6](#_Toc135167362)

[2.Risk of bias 8](#_Toc135167363)

[3.Funnel plot 9](#_Toc135167364)

# 1.Search strategy

## 1.1 PubMed

| #1 | (Child[MeSH Terms]) OR (Children[Title/Abstract]) |
| --- | --- |
| #2 | Adolescent[MeSH Terms] OR ((((((((((((((((Adolescents[Title/Abstract]) OR (Adolescence[Title/Abstract])) OR (Teens[Title/Abstract])) OR (Teen[Title/Abstract])) OR (Teenagers[Title/Abstract])) OR (Teenager[Title/Abstract])) OR (Youth[Title/Abstract])) OR (Youths[Title/Abstract])) OR (Adolescents, Female[Title/Abstract])) OR (Adolescent, Female[Title/Abstract])) OR (Female Adolescent[Title/Abstract])) OR (Female Adolescents[Title/Abstract])) OR (Adolescents, Male[Title/Abstract])) OR (Adolescent, Male[Title/Abstract])) OR (Male Adolescent[Title/Abstract])) OR (Male Adolescents[Title/Abstract])) |
| #3 | ((((((((((((((((((((((((((((((((((((((((((Arthritis, Juvenile[MeSH Terms]) OR (Juvenile Arthritis[Title/Abstract])) OR (Childhood Arthritis[Title/Abstract])) OR (Arthritides, Childhood[Title/Abstract])) OR (Arthritis, Childhood[Title/Abstract])) OR (Childhood Arthritides[Title/Abstract])) OR (Arthritis, Juvenile Chronic[Title/Abstract])) OR (Juvenile Chronic Arthritis[Title/Abstract])) OR (Chronic Arthritis, Juvenile[Title/Abstract])) OR (Juvenile Idiopathic Arthritis[Title/Abstract])) OR (Idiopathic Arthritis, Juvenile[Title/Abstract])) OR (Arthritis, Juvenile Idiopathic[Title/Abstract])) OR (Juvenile Rheumatoid Arthritis[Title/Abstract])) OR (Arthritis, Juvenile Rheumatoid[Title/Abstract])) OR (Rheumatoid Arthritis, Juvenile[Title/Abstract])) OR (Oligoarthritis, Juvenile[Title/Abstract])) OR (Juvenile Oligoarthritis[Title/Abstract])) OR (Psoriatic Arthritis, Juvenile[Title/Abstract])) OR (Arthritis, Juvenile Psoriatic[Title/Abstract])) OR (Juvenile Psoriatic Arthritis[Title/Abstract])) OR (Enthesitis-Related Arthritis, Juvenile[Title/Abstract])) OR (Arthritis, Juvenile Enthesitis-Related[Title/Abstract])) OR (Enthesitis Related Arthritis, Juvenile[Title/Abstract])) OR (Juvenile Enthesitis-Related Arthritis[Title/Abstract])) OR (Polyarthritis, Juvenile, Rheumatoid Factor Negative[Title/Abstract])) OR (Juvenile-Onset Still Disease[Title/Abstract])) OR (Juvenile Onset Still Disease[Title/Abstract])) OR (Still Disease, Juvenile-Onset[Title/Abstract])) OR (Still Disease, Juvenile Onset[Title/Abstract])) OR (Still's Disease, Juvenile-Onset[Title/Abstract])) OR (Juvenile-Onset Still's Disease[Title/Abstract])) OR (Still's Disease, Juvenile Onset[Title/Abstract])) OR (Systemic Arthritis, Juvenile[Title/Abstract])) OR (Arthritis, Juvenile Systemic[Title/Abstract])) OR (Juvenile Systemic Arthritis[Title/Abstract])) OR (Juvenile-Onset Stills Disease[Title/Abstract])) OR (Juvenile Onset Stills Disease[Title/Abstract])) OR (Stills Disease, Juvenile-Onset[Title/Abstract])) OR (Polyarthritis, Juvenile, Rheumatoid Factor Positive[Title/Abstract])) OR (Polyarticular Juvenile Idiopathic Arthritis[Title/Abstract])) OR (Polyarticular-Course Juvenile Idiopathic Arthritis[Title/Abstract])) OR (PCJIA[Title/Abstract])) OR (PJIA Polyarticular Juvenile Idiopathic Arthritis[Title/Abstract]) |
| #4 | (Exercise[MeSH Terms]) or (((((((((((((((((((((Physical Activity[Title/Abstract]) OR (Activities, Physical[Title/Abstract])) OR (Activity, Physical[Title/Abstract])) OR (Physical Activities[Title/Abstract])) OR (Exercise, Physical[Title/Abstract])) OR (Exercises, Physical[Title/Abstract])) OR (Physical Exercise[Title/Abstract])) OR (Physical Exercises[Title/Abstract])) OR (Exercise, Isometric[Title/Abstract])) OR (Exercises, Isometric[Title/Abstract])) OR (Isometric Exercises[Title/Abstract])) OR (Isometric Exercise[Title/Abstract])) OR (Exercise, Aerobic[Title/Abstract])) OR (Aerobic Exercise[Title/Abstract])) OR (Aerobic Exercises[Title/Abstract])) OR (Exercises, Aerobic[Title/Abstract])) OR (Exercise Training[Title/Abstract])) OR (Exercise Trainings[Title/Abstract])) OR (Training, Exercise[Title/Abstract])) OR (Trainings, Exercise[Title/Abstract] OR (taichi[Title/Abstract])OR (yoga[Title/Abstract]) OR (pilates[Title/Abstract]) OR (walk[Title/Abstract]) OR (walking[Title/Abstract]) OR (weight[Title/Abstract])))) |
| #5 | ((Child[MeSH Terms]) OR (Children[Title/Abstract])) OR (Adolescent[MeSH Terms] OR ((((((((((((((((Adolescents[Title/Abstract]) OR (Adolescence[Title/Abstract])) OR (Teens[Title/Abstract])) OR (Teen[Title/Abstract])) OR (Teenagers[Title/Abstract])) OR (Teenager[Title/Abstract])) OR (Youth[Title/Abstract])) OR (Youths[Title/Abstract])) OR (Adolescents, Female[Title/Abstract])) OR (Adolescent, Female[Title/Abstract])) OR (Female Adolescent[Title/Abstract])) OR (Female Adolescents[Title/Abstract])) OR (Adolescents, Male[Title/Abstract])) OR (Adolescent, Male[Title/Abstract])) OR (Male Adolescent[Title/Abstract])) OR (Male Adolescents[Title/Abstract]))) |
| #6 | randomized controlled trial[Publication Type] OR randomized[Title/Abstract] OR placebo[Title/Abstract] |
| #7 | (((((Child[MeSH Terms]) OR (Children[Title/Abstract])) OR (Adolescent[MeSH Terms] OR ((((((((((((((((Adolescents[Title/Abstract]) OR (Adolescence[Title/Abstract])) OR (Teens[Title/Abstract])) OR (Teen[Title/Abstract])) OR (Teenagers[Title/Abstract])) OR (Teenager[Title/Abstract])) OR (Youth[Title/Abstract])) OR (Youths[Title/Abstract])) OR (Adolescents, Female[Title/Abstract])) OR (Adolescent, Female[Title/Abstract])) OR (Female Adolescent[Title/Abstract])) OR (Female Adolescents[Title/Abstract])) OR (Adolescents, Male[Title/Abstract])) OR (Adolescent, Male[Title/Abstract])) OR (Male Adolescent[Title/Abstract])) OR (Male Adolescents[Title/Abstract])))) AND (((((((((((((((((((((((((((((((((((((((((((Arthritis, Juvenile[MeSH Terms]) OR (Juvenile Arthritis[Title/Abstract])) OR (Childhood Arthritis[Title/Abstract])) OR (Arthritides, Childhood[Title/Abstract])) OR (Arthritis, Childhood[Title/Abstract])) OR (Childhood Arthritides[Title/Abstract])) OR (Arthritis, Juvenile Chronic[Title/Abstract])) OR (Juvenile Chronic Arthritis[Title/Abstract])) OR (Chronic Arthritis, Juvenile[Title/Abstract])) OR (Juvenile Idiopathic Arthritis[Title/Abstract])) OR (Idiopathic Arthritis, Juvenile[Title/Abstract])) OR (Arthritis, Juvenile Idiopathic[Title/Abstract])) OR (Juvenile Rheumatoid Arthritis[Title/Abstract])) OR (Arthritis, Juvenile Rheumatoid[Title/Abstract])) OR (Rheumatoid Arthritis, Juvenile[Title/Abstract])) OR (Oligoarthritis, Juvenile[Title/Abstract])) OR (Juvenile Oligoarthritis[Title/Abstract])) OR (Psoriatic Arthritis, Juvenile[Title/Abstract])) OR (Arthritis, Juvenile Psoriatic[Title/Abstract])) OR (Juvenile Psoriatic Arthritis[Title/Abstract])) OR (Enthesitis-Related Arthritis, Juvenile[Title/Abstract])) OR (Arthritis, Juvenile Enthesitis-Related[Title/Abstract])) OR (Enthesitis Related Arthritis, Juvenile[Title/Abstract])) OR (Juvenile Enthesitis-Related Arthritis[Title/Abstract])) OR (Polyarthritis, Juvenile, Rheumatoid Factor Negative[Title/Abstract])) OR (Juvenile-Onset Still Disease[Title/Abstract])) OR (Juvenile Onset Still Disease[Title/Abstract])) OR (Still Disease, Juvenile-Onset[Title/Abstract])) OR (Still Disease, Juvenile Onset[Title/Abstract])) OR (Still's Disease, Juvenile-Onset[Title/Abstract])) OR (Juvenile-Onset Still's Disease[Title/Abstract])) OR (Still's Disease, Juvenile Onset[Title/Abstract])) OR (Systemic Arthritis, Juvenile[Title/Abstract])) OR (Arthritis, Juvenile Systemic[Title/Abstract])) OR (Juvenile Systemic Arthritis[Title/Abstract])) OR (Juvenile-Onset Stills Disease[Title/Abstract])) OR (Juvenile Onset Stills Disease[Title/Abstract])) OR (Stills Disease, Juvenile-Onset[Title/Abstract])) OR (Polyarthritis, Juvenile, Rheumatoid Factor Positive[Title/Abstract])) OR (Polyarticular Juvenile Idiopathic Arthritis[Title/Abstract])) OR (Polyarticular-Course Juvenile Idiopathic Arthritis[Title/Abstract])) OR (PCJIA[Title/Abstract])) OR (PJIA Polyarticular Juvenile Idiopathic Arthritis[Title/Abstract]))) AND ((Exercise[MeSH Terms]) or (((((((((((((((((((((Physical Activity[Title/Abstract]) OR (Activities, Physical[Title/Abstract])) OR (Activity, Physical[Title/Abstract])) OR (Physical Activities[Title/Abstract])) OR (Exercise, Physical[Title/Abstract])) OR (Exercises, Physical[Title/Abstract])) OR (Physical Exercise[Title/Abstract])) OR (Physical Exercises[Title/Abstract])) OR (Exercise, Isometric[Title/Abstract])) OR (Exercises, Isometric[Title/Abstract])) OR (Isometric Exercises[Title/Abstract])) OR (Isometric Exercise[Title/Abstract])) OR (Exercise, Aerobic[Title/Abstract])) OR (Aerobic Exercise[Title/Abstract])) OR (Aerobic Exercises[Title/Abstract])) OR (Exercises, Aerobic[Title/Abstract])) OR (Exercise Training[Title/Abstract])) OR (Exercise Trainings[Title/Abstract])) OR (Training, Exercise[Title/Abstract])) OR (Trainings, Exercise[Title/Abstract] OR (taichi[Title/Abstract])OR (yoga[Title/Abstract]) OR (pilates[Title/Abstract]) OR (walk[Title/Abstract]) OR (walking[Title/Abstract]) OR (weight[Title/Abstract])))))) AND (randomized controlled trial[Publication Type] OR randomized[Title/Abstract] OR placebo[Title/Abstract]) |

## 1.2 Embase

| #1 | 'exercise'/exp OR 'exercise' |
| --- | --- |
| #2 | 'physical activity':ab,ti OR 'activities, physical':ab,ti OR 'activity, physical':ab,ti OR 'physical activities':ab,ti OR 'exercise, physical':ab,ti OR 'exercises, physical':ab,ti OR 'physical exercise':ab,ti OR 'physical exercises':ab,ti OR 'exercise, isometric':ab,ti OR 'exercises, isometric':ab,ti OR 'isometric exercises':ab,ti OR 'isometric exercise':ab,ti OR 'exercise aerobic':ab,ti OR 'aerobic exercise':ab,ti OR 'aerobic exercises':ab,ti OR 'exercises, aerobic':ab,ti OR 'exercise training':ab,ti OR 'exercise trainings':ab,ti OR 'training, exercise':ab,ti OR 'trainings, exercise':ab,ti OR 'taichi':ab,ti OR 'yoga':ab,ti OR 'pilates':ab,ti OR 'walk':ab,ti OR 'walking':ab,ti OR 'weight':ab,ti |
| #3 | 'child'/exp |
| #4 | 'children':ab,ti |
| #5 | 'adolescent'/exp |
| #6 | 'adolescents':ab,ti OR 'adolescence':ab,ti OR 'teens':ab,ti OR 'teen':ab,ti OR 'teenagers':ab,ti OR 'teenager':ab,ti OR 'youth':ab,ti OR 'youths':ab,ti OR 'adolescents, female':ab,ti OR 'adolescent, female':ab,ti OR 'female adolescent':ab,ti OR 'female adolescents':ab,ti OR 'adolescents, male':ab,ti OR 'adolescent, male':ab,ti OR 'male adolescent':ab,ti OR 'male adolescents':ab,ti |
| #7 | 'arthritis, juvenile'/exp |
| #8 | 'juvenile arthritis':ab,ti OR 'childhood arthritis':ab,ti OR 'arthritides, childhood':ab,ti OR 'arthritis, childhood':ab,ti OR 'childhood arthritides':ab,ti OR 'arthritis, juvenile chronic':ab,ti OR 'juvenile chronic arthritis':ab,ti OR 'chronic arthritis, juvenile':ab,ti OR 'juvenile idiopathic arthritis':ab,ti OR 'idiopathic arthritis, juvenile':ab,ti OR 'arthritis, juvenile idiopathic':ab,ti OR 'juvenile rheumatoid arthritis':ab,ti OR 'arthritis, juvenile rheumatoid':ab,ti OR 'rheumatoid arthritis, juvenile':ab,ti OR 'oligoarthritis, juvenile':ab,ti OR 'juvenile oligoarthritis':ab,ti OR 'psoriatic arthritis, juvenile':ab,ti OR 'arthritis, juvenile psoriatic':ab,ti OR 'juvenile psoriatic arthritis':ab,ti OR 'enthesitis-related arthritis, juvenile':ab,ti OR 'arthritis, juvenile enthesitis-related':ab,ti OR 'enthesitis related arthritis, juvenile':ab,ti OR 'juvenile enthesitis-related arthritis':ab,ti OR 'polyarthritis, juvenile, rheumatoid factor  negative':ab,ti OR 'juvenile-onset still disease':ab,ti OR 'juvenile onset still disease':ab,ti OR 'still disease, juvenile-onset':ab,ti OR 'still disease, juvenile onset':ab,ti OR 'stills disease, juvenile onset':ab,ti OR 'systemic arthritis, juvenile':ab,ti OR 'arthritis, juvenile systemic':ab,ti OR 'juvenile systemic arthritis':ab,ti OR 'juvenile-onset stills disease':ab,ti OR 'juvenile onset stills disease':ab,ti OR 'stills disease, juvenile-onset':ab,ti OR 'polyarthritis, juvenile, rheumatoid factor positive':ab,ti OR 'polyarticular juvenile idiopathic arthritis':ab,ti OR 'polyarticular-course juvenile idiopathic arthritis':ab,ti OR 'pcjia':ab,ti OR 'pjia polyarticular juvenile idiopathic arthritis':ab,ti |
| #9 | 'randomized controlled trial':ab,ti OR 'randomized':ab,ti OR 'placebo':ab,ti |
| #10 | #1 OR #2 |
| #11 | #3 OR #4 OR #5 OR #6 |
| #12 | #7 OR #8 |
| #13 | #9 AND #10 AND #11 AND #12 |

## 1.3 Web of Science

TS=(Exercise OR Exercises OR Physical Activity OR Activities, Physical OR Activity, Physical OR Physical Activities OR Exercise, Physical OR Exercises, Physical OR Physical Exercise OR Physical Exercises OR Acute Exercise OR Acute Exercises OR Exercise, Acute OR Exercises, Acute OR Exercise, Isometric OR Exercises, Isometric OR Isometric Exercises OR Isometric Exercise OR Exercise, Aerobic OR Aerobic Exercise OR Aerobic Exercises OR Exercises, Aerobic OR Exercise Training OR Exercise Trainings OR Training, Exercise OR Trainings, Exercise OR taichi OR yoga OR Pilates OR walk OR weight) AND TS=(Child OR Children OR Adolescent OR Adolescents OR Adolescence OR Teens OR Teen OR Teenagers OR Teenager OR Youth OR Youths OR Adolescents, Female OR Adolescent, Female OR Female Adolescent OR Female Adolescents OR Adolescents, Male OR Adolescent, Male OR Male Adolescent OR Male Adolescents) AND TS=(Arthritis, Juvenile OR Juvenile Arthritis OR Childhood Arthritis OR Arthritides, Childhood OR Arthritis, Childhood OR Childhood Arthritides OR Arthritis, Juvenile Chronic OR Juvenile Chronic Arthritis OR Chronic Arthritis, Juvenile OR Juvenile Idiopathic Arthritis OR Idiopathic Arthritis, Juvenile OR Arthritis, Juvenile Idiopathic OR Juvenile Rheumatoid Arthritis OR Arthritis, Juvenile Rheumatoid OR Rheumatoid Arthritis, Juvenile OR Oligoarthritis, Juvenile OR Juvenile Oligoarthritis OR Psoriatic Arthritis, Juvenile OR Arthritis, Juvenile Psoriatic OR Juvenile Psoriatic Arthritis OR Enthesitis-Related Arthritis, Juvenile OR Arthritis, Juvenile Enthesitis-Related OR Enthesitis Related Arthritis, Juvenile OR Juvenile Enthesitis-Related Arthritis OR Polyarthritis, Juvenile, Rheumatoid Factor Negative OR Juvenile-Onset Still Disease OR Juvenile Onset Still Disease OR Still Disease, Juvenile-Onset OR Still Disease, Juvenile Onset OR Still's Disease, Juvenile-Onset OR Juvenile-Onset Still's Disease OR Still's Disease, Juvenile Onset OR Systemic Arthritis, Juvenile OR Arthritis, Juvenile Systemic OR Juvenile Systemic Arthritis OR Juvenile-Onset Stills Disease OR Juvenile Onset Stills Disease OR Stills Disease, Juvenile-Onset OR Polyarthritis, Juvenile, Rheumatoid Factor Positive OR Polyarticular Juvenile Idiopathic Arthritis OR Polyarticular-Course Juvenile Idiopathic Arthritis OR PCJIA OR PJIA Polyarticular Juvenile Idiopathic Arthritis) AND TS=(randomized controlled trial OR randomized OR placebo)

## 1.4 Cochrane library

| #1 | MeSH descriptor: [Child] explode all trees |
| --- | --- |
| #2 | (Children):ti,ab,kw |
| #3 | MeSH descriptor: [Adolescent] explode all trees |
| #4 | (Adolescents):ti,ab,kw OR (Adolescence):ti,ab,kw OR (Teens):ti,ab,kw OR (Teen):ti,ab,kw OR (Teenagers):ti,ab,kw OR (Teenager):ti,ab,kw OR (Youth):ti,ab,kw OR (Youths):ti,ab,kw OR (Adolescents, Female):ti,ab,kw OR (Adolescent, Female):ti,ab,kw OR (Female Adolescent):ti,ab,kw OR (Female Adolescents):ti,ab,kw OR (Adolescents, Male):ti,ab,kw OR (Adolescent, Male):ti,ab,kw OR (Male Adolescent):ti,ab,kw OR (Male Adolescents):ti,ab,kw |
| #5 | MeSH descriptor: [Exercise] explode all trees |
| #6 | (Exercises):ti,ab,kw OR (Physical Activity):ti,ab,kw OR (Activities, Physical):ti,ab,kw OR (Activity, Physical):ti,ab,kw OR (Physical Activities):ti,ab,kw OR (Exercise, Physical):ti,ab,kw OR (Exercises, Physical):ti,ab,kw OR (Physical Exercise):ti,ab,kw OR (Physical Exercises):ti,ab,kw OR (Acute Exercise):ti,ab,kw OR (Acute Exercises):ti,ab,kw OR (Exercise, Acute):ti,ab,kw OR (Exercises, Acute):ti,ab,kw OR (Exercise, Isometric):ti,ab,kw OR (Exercises, Isometric):ti,ab,kw OR (Isometric Exercises):ti,ab,kw OR (Isometric Exercise):ti,ab,kw OR (Exercise, Aerobic):ti,ab,kw OR (Aerobic Exercise):ti,ab,kw OR (Aerobic Exercises):ti,ab,kw OR (Exercises, Aerobic):ti,ab,kw OR (Exercise Training):ti,ab,kw OR (Exercise Trainings):ti,ab,kw OR (Training, Exercise):ti,ab,kw OR (Trainings, Exercise):ti,ab,kw OR (taichi):ti,ab,kw OR (yoga):ti,ab,kw OR (pilates):ti,ab,kw OR (walk):ti,ab,kw OR (walking):ti,ab,kw OR (weight):ti,ab,kw |
| #7 | MeSH descriptor: [Arthritis, Juvenile] explode all trees |
| #8 | (Juvenile Arthritis):ti,ab,kw OR (Childhood Arthritis):ti,ab,kw OR (Arthritides, Childhood):ti,ab,kw OR (Arthritis, Childhood):ti,ab,kw OR (Childhood Arthritides):ti,ab,kw OR (Arthritis, Juvenile Chronic):ti,ab,kw OR (Juvenile Chronic Arthritis):ti,ab,kw OR (Chronic Arthritis, Juvenile):ti,ab,kw OR (Juvenile Idiopathic Arthritis):ti,ab,kw OR (Idiopathic Arthritis, Juvenile):ti,ab,kw OR (Arthritis, Juvenile Idiopathic):ti,ab,kw OR (Juvenile Rheumatoid Arthritis):ti,ab,kw OR (Arthritis, Juvenile Rheumatoid):ti,ab,kw OR (Rheumatoid Arthritis, Juvenile):ti,ab,kw OR (Oligoarthritis, Juvenile):ti,ab,kw OR (Juvenile Oligoarthritis):ti,ab,kw OR (Psoriatic Arthritis, Juvenile):ti,ab,kw OR (Arthritis, Juvenile Psoriatic):ti,ab,kw OR (Juvenile Psoriatic Arthritis):ti,ab,kw OR (Enthesitis-Related Arthritis, Juvenile):ti,ab,kw OR (Arthritis, Juvenile Enthesitis-Related):ti,ab,kw OR (Enthesitis Related Arthritis, Juvenile):ti,ab,kw OR (Juvenile Enthesitis-Related Arthritis):ti,ab,kw OR (Polyarthritis, Juvenile, Rheumatoid Factor Negative):ti,ab,kw OR (Juvenile-Onset Still Disease):ti,ab,kw OR (Juvenile Onset Still Disease):ti,ab,kw OR (Still Disease, Juvenile-Onset):ti,ab,kw OR (Still Disease, Juvenile Onset):ti,ab,kw OR (Still's Disease, Juvenile-Onset):ti,ab,kw OR (Juvenile-Onset Still's Disease):ti,ab,kw OR (Still's Disease, Juvenile Onset):ti,ab,kw OR (Systemic Arthritis, Juvenile):ti,ab,kw OR (Arthritis, Juvenile Systemic):ti,ab,kw OR (Juvenile Systemic Arthritis):ti,ab,kw OR (Juvenile-Onset Stills Disease):ti,ab,kw OR (Juvenile Onset Stills Disease):ti,ab,kw OR (Stills Disease, Juvenile-Onset):ti,ab,kw OR (Polyarthritis, Juvenile, Rheumatoid Factor Positive):ti,ab,kw OR (Polyarticular Juvenile Idiopathic Arthritis):ti,ab,kw OR (Polyarticular-Course Juvenile Idiopathic Arthritis):ti,ab,kw OR (PCJIA):ti,ab,kw OR (PJIA Polyarticular Juvenile Idiopathic Arthritis):ti,ab,kw |
| #9 | #1 OR #2 OR #3 OR #4 |
| #10 | #5 OR #6 |
| #11 | #7 OR #8 |
| #12 | #9 AND #10 AND #11 |

# 2.Risk of bias

**
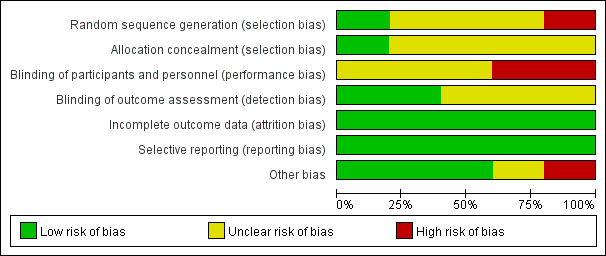
**

**Figure S1.** Risk of bias summary


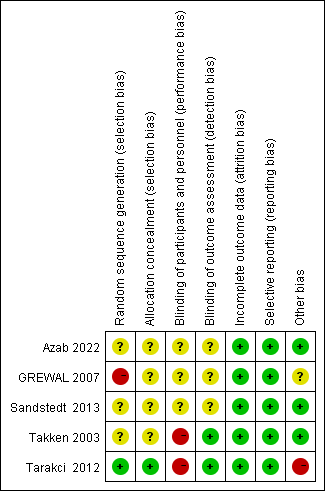


**Figure S2.** Risk of bias detail

# 3.Funnel plot


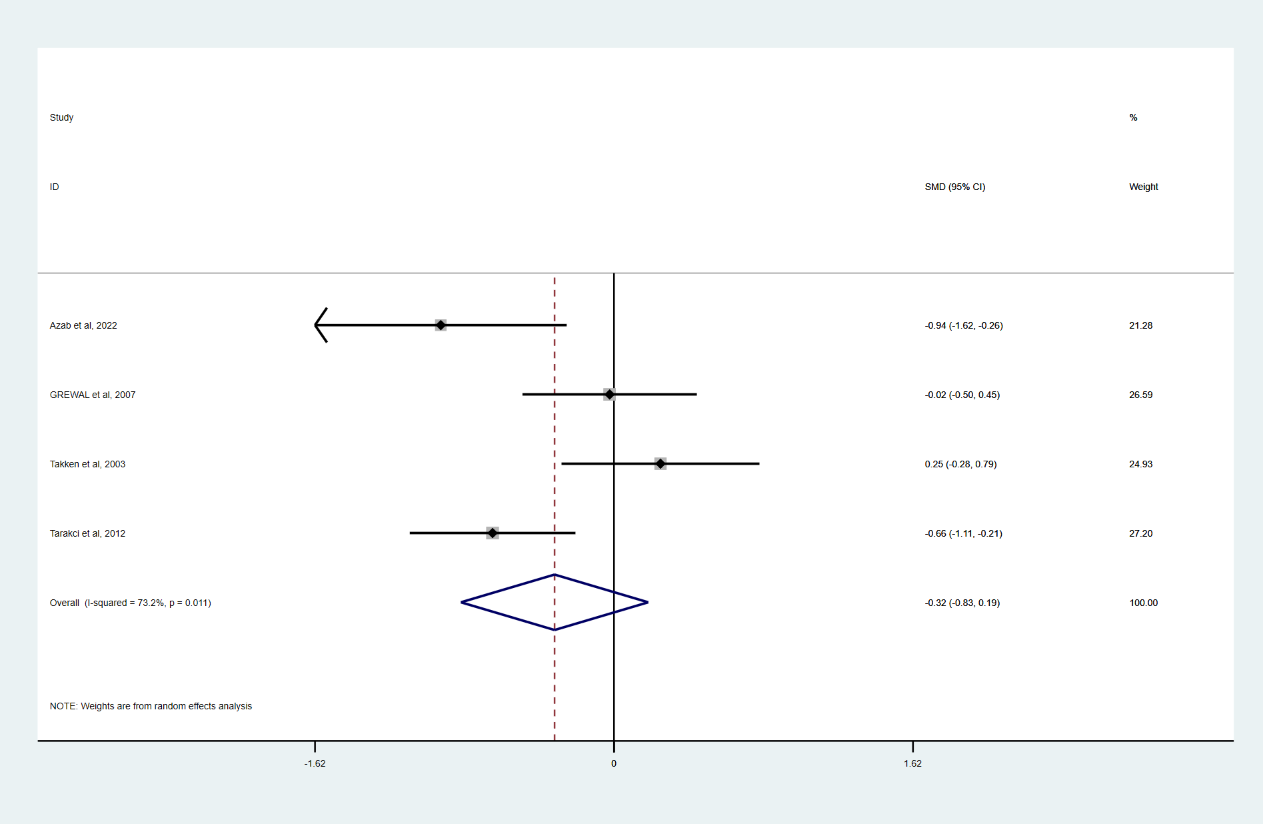
**Figure S3.** The funnel plot of Childhood Health Assessment Questionnaire


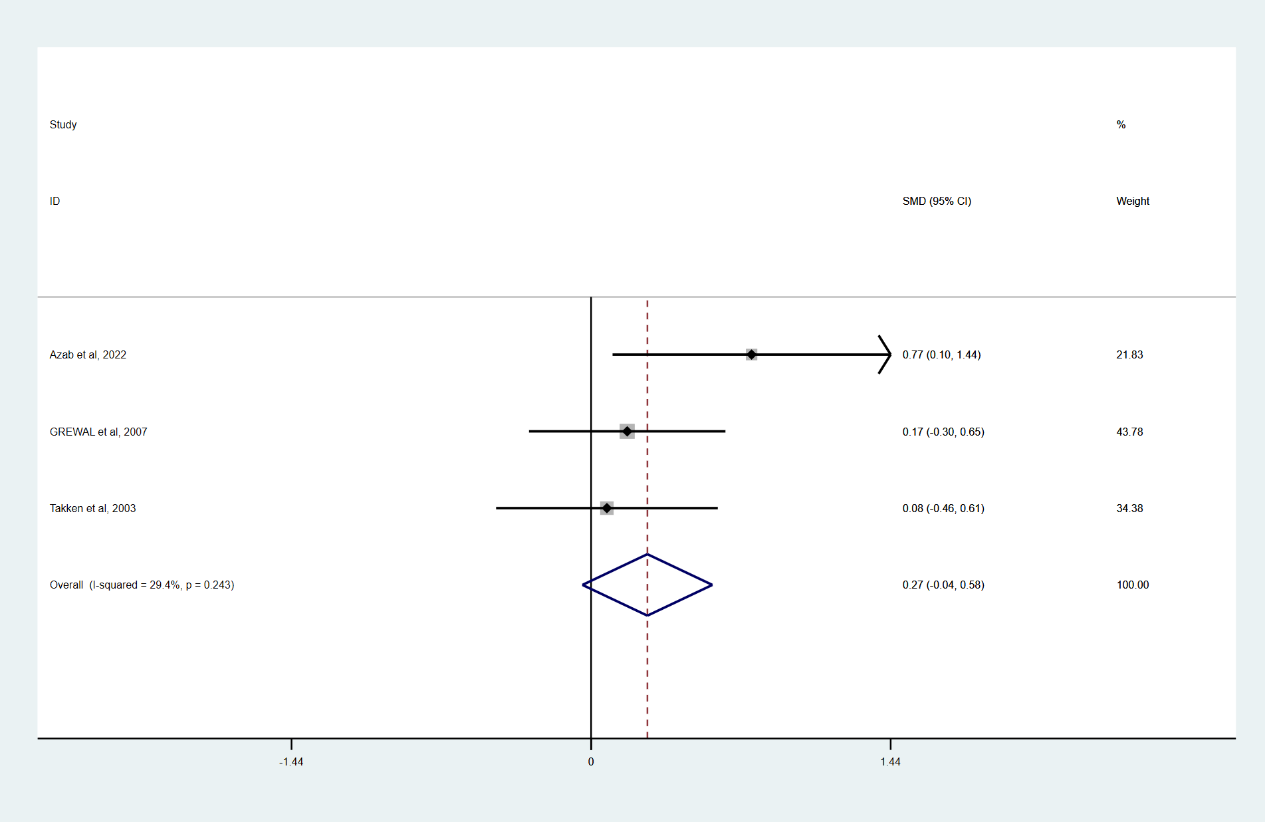


**Figure S4.** The funnel plot of Quality of Life


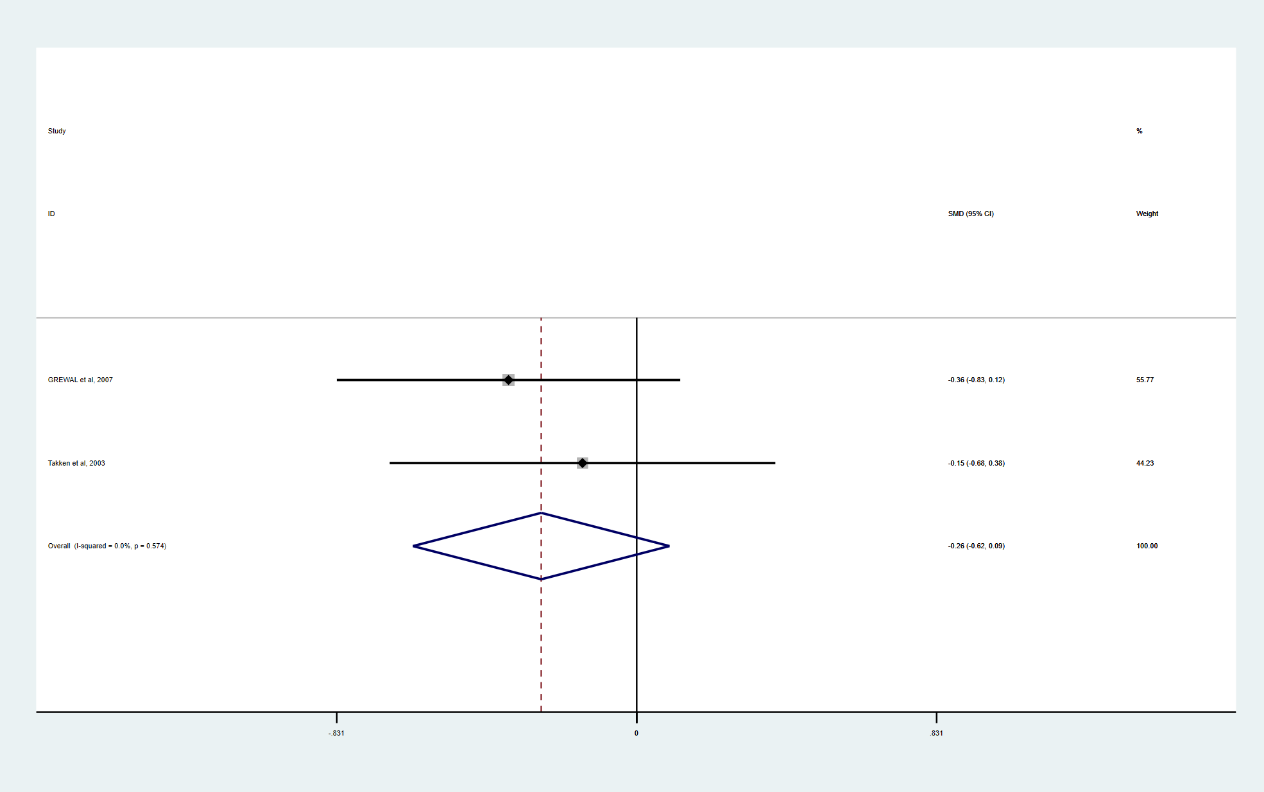


**Figure S5.** The funnel plot of Pediatric Escola Paulista de Medicina Range of Motion scale


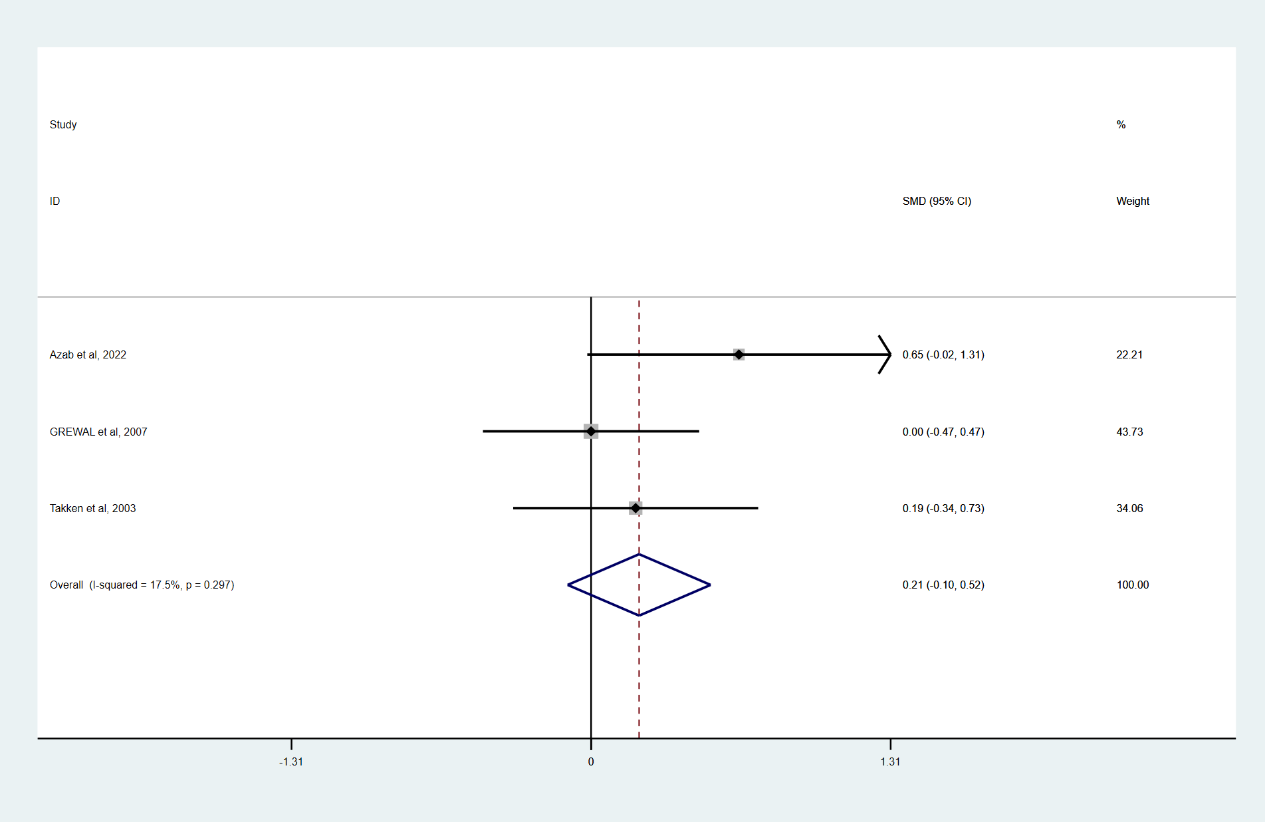


**Figure S6.** The funnel plot of peak oxygen consumption


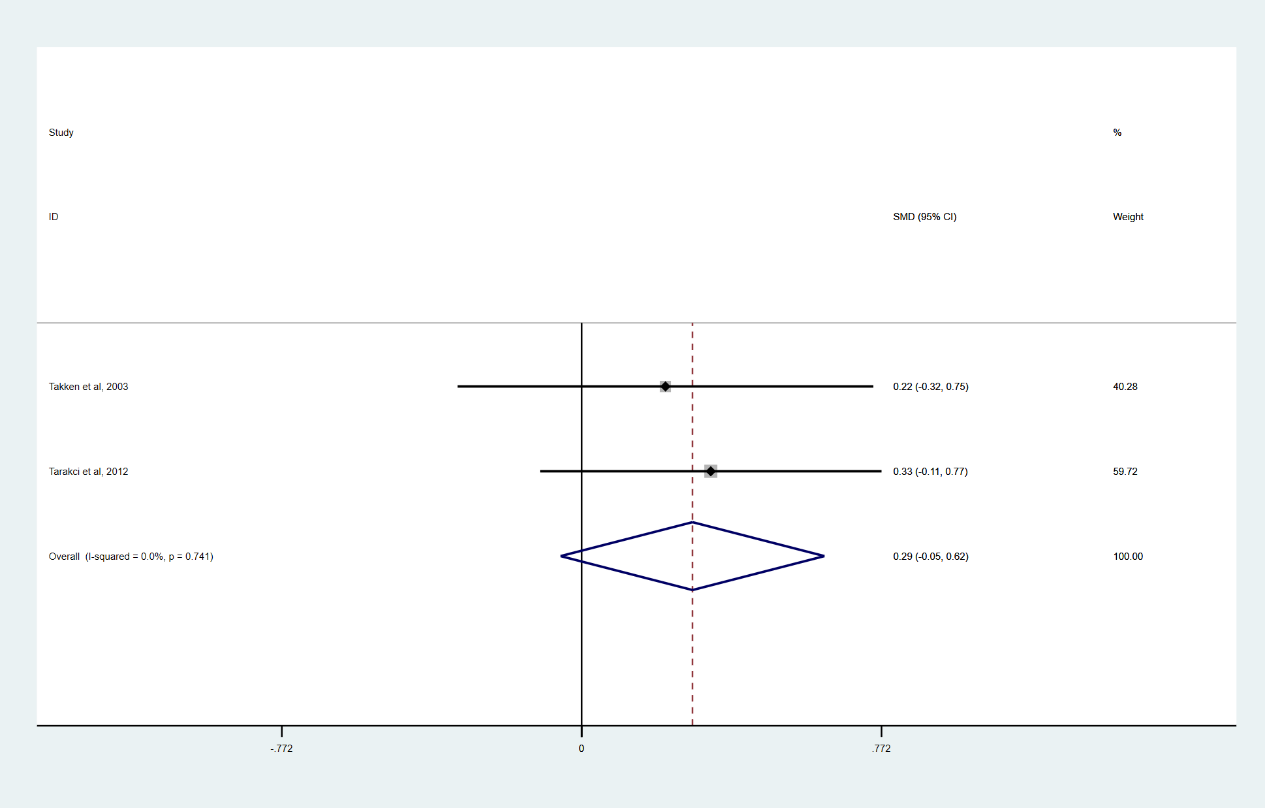


**Figure S7.** The funnel plot of Six-minute walk test


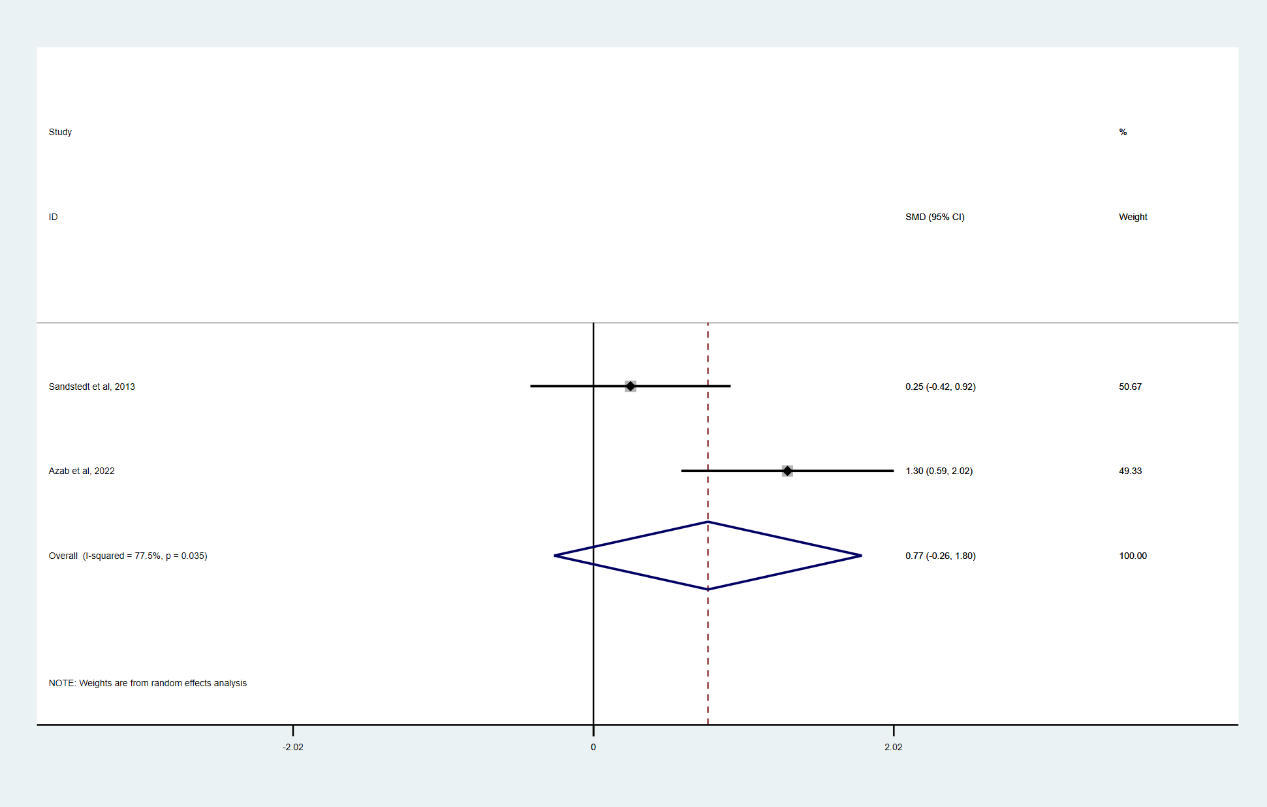


**Figure S8.** The funnel plot of max heart rate


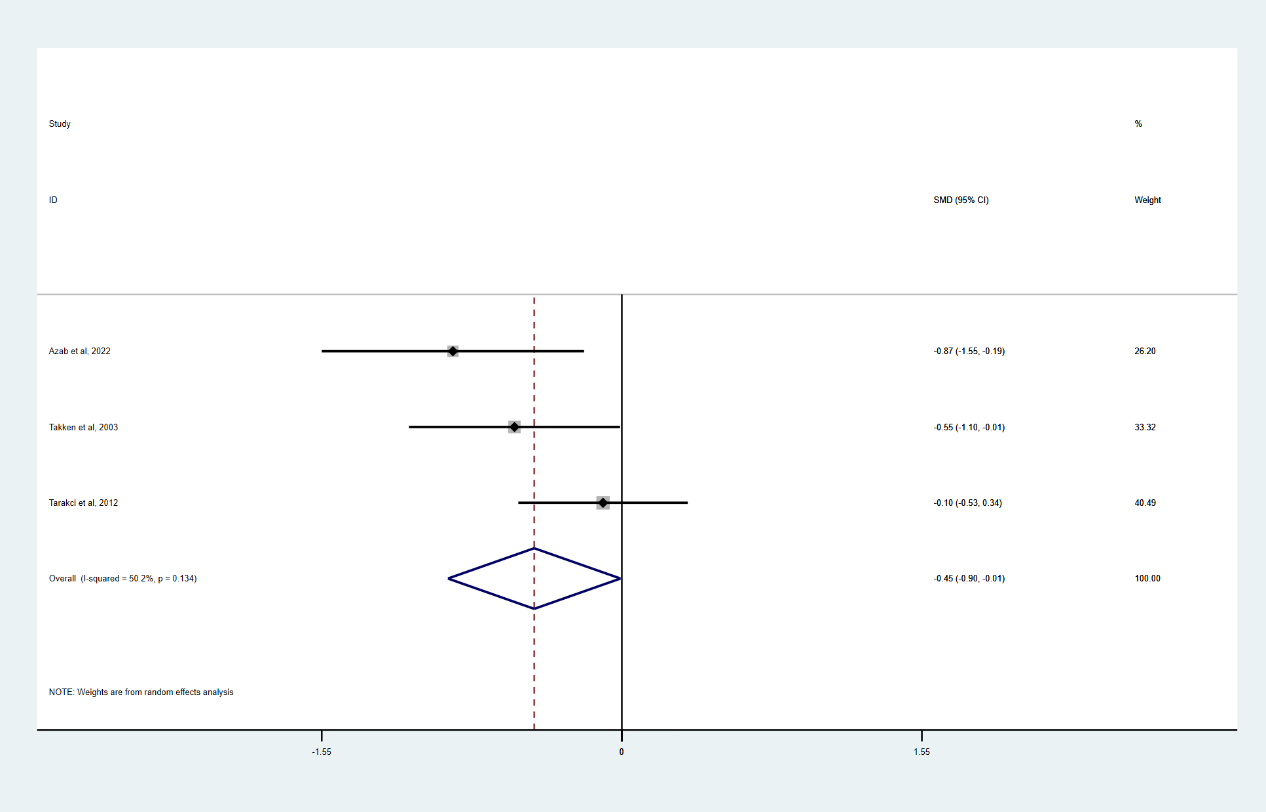


**Figure S9.** The funnel plot of pain

# **4.**Sensitivity analysis


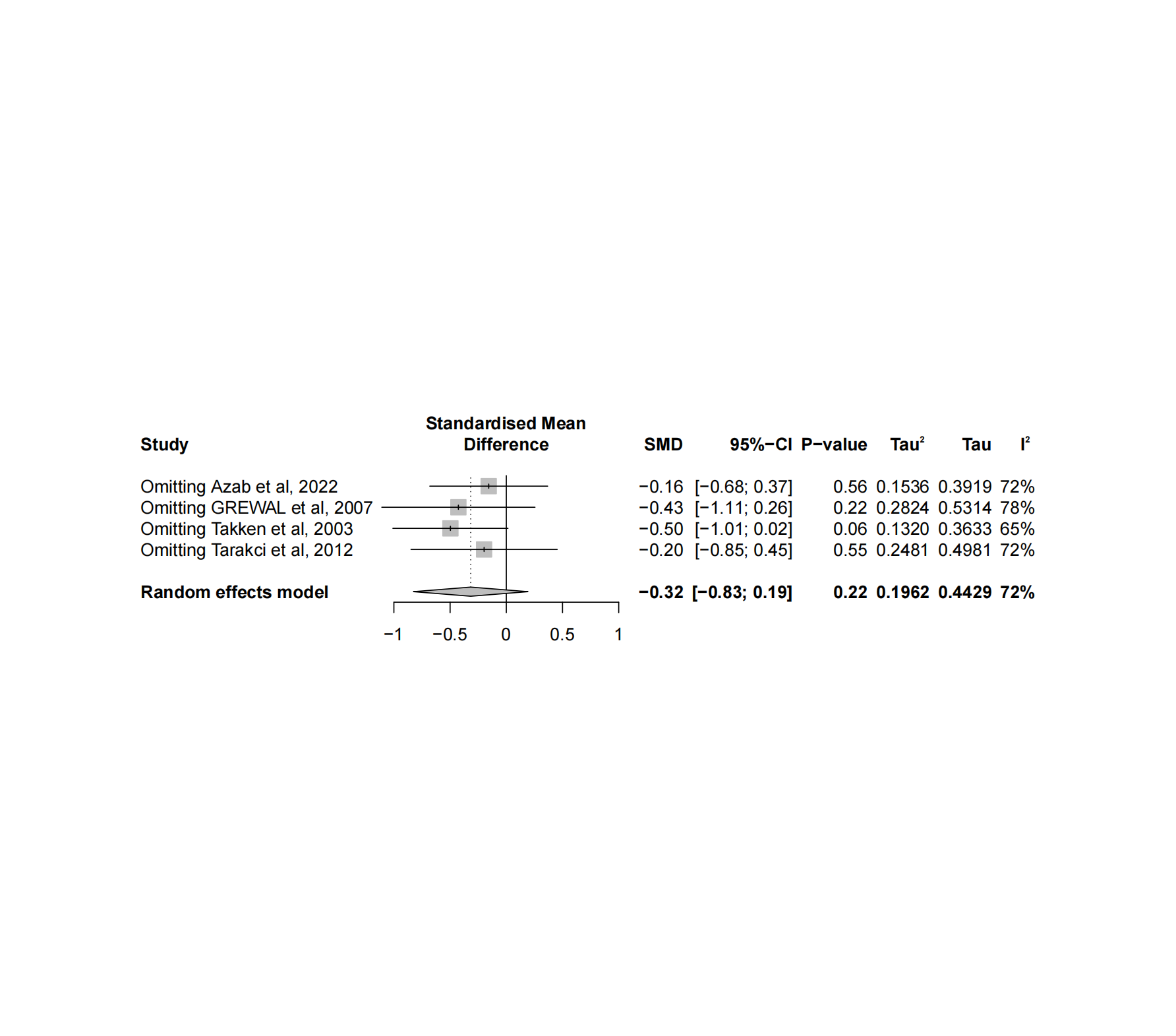


**Figure S10.** Sensitivity analysis of CHAQ


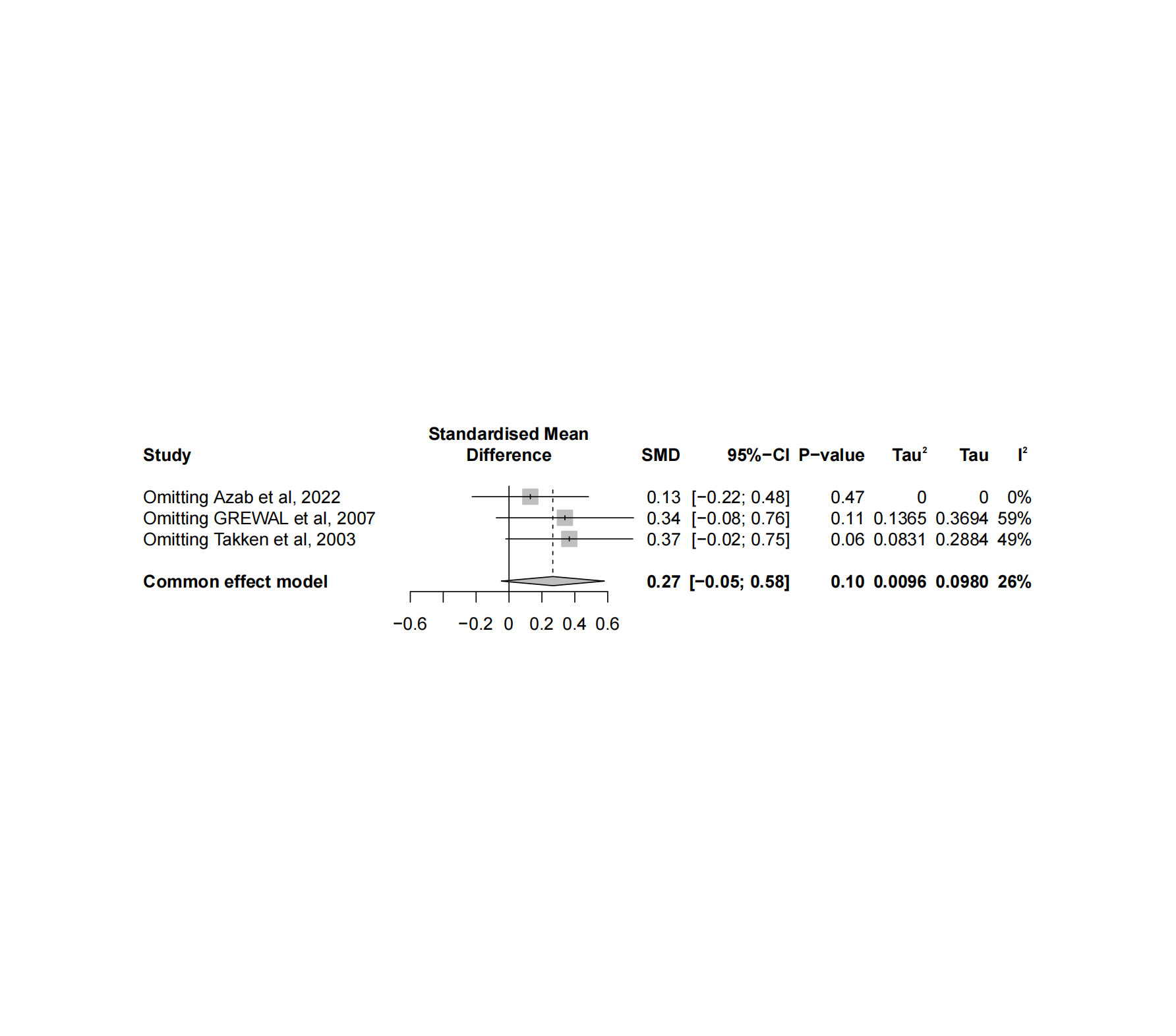


**Figure S11.** Sensitivity analysis of QoL


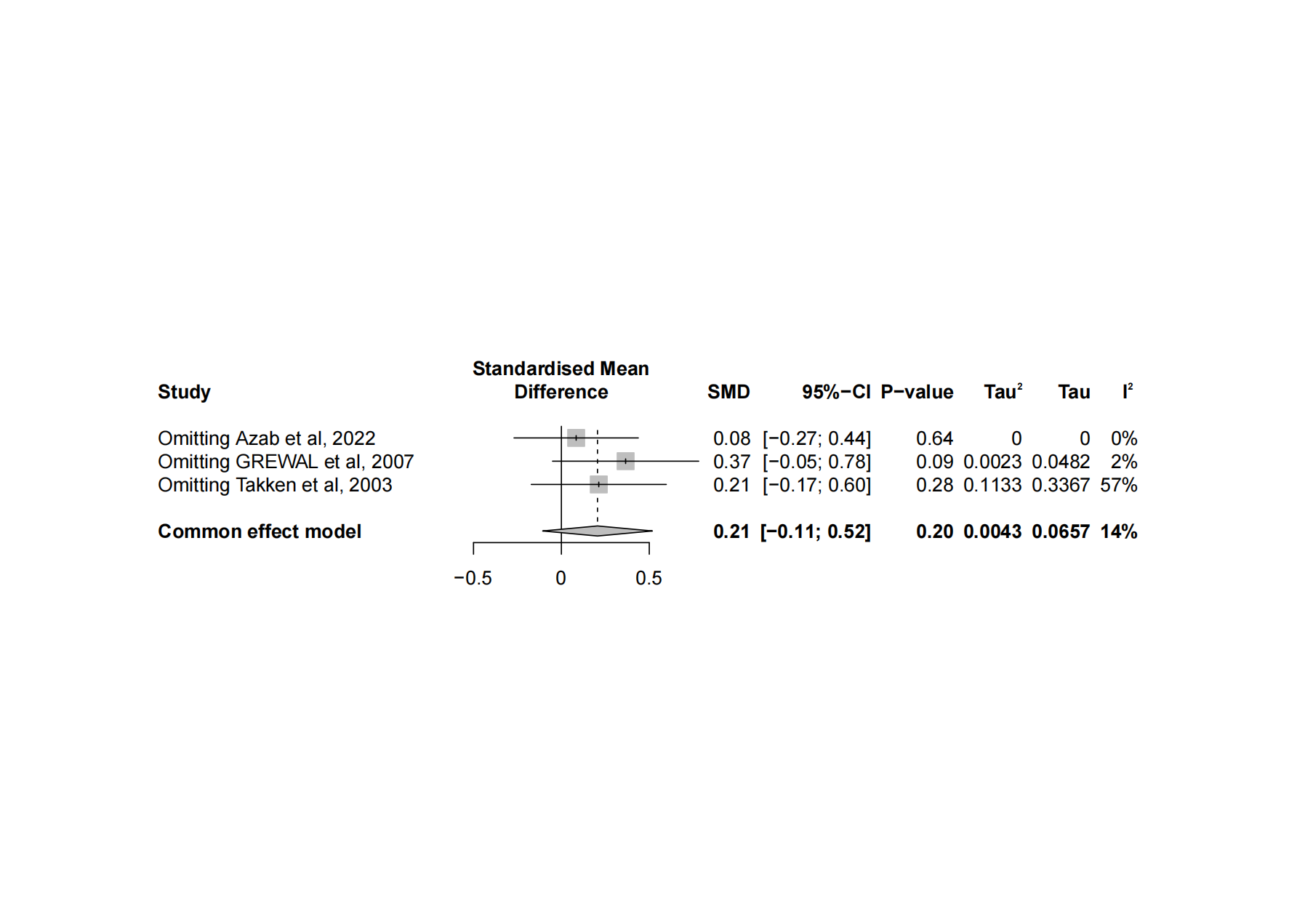


**Figure S12.** Sensitivity analysis of VO_2_max


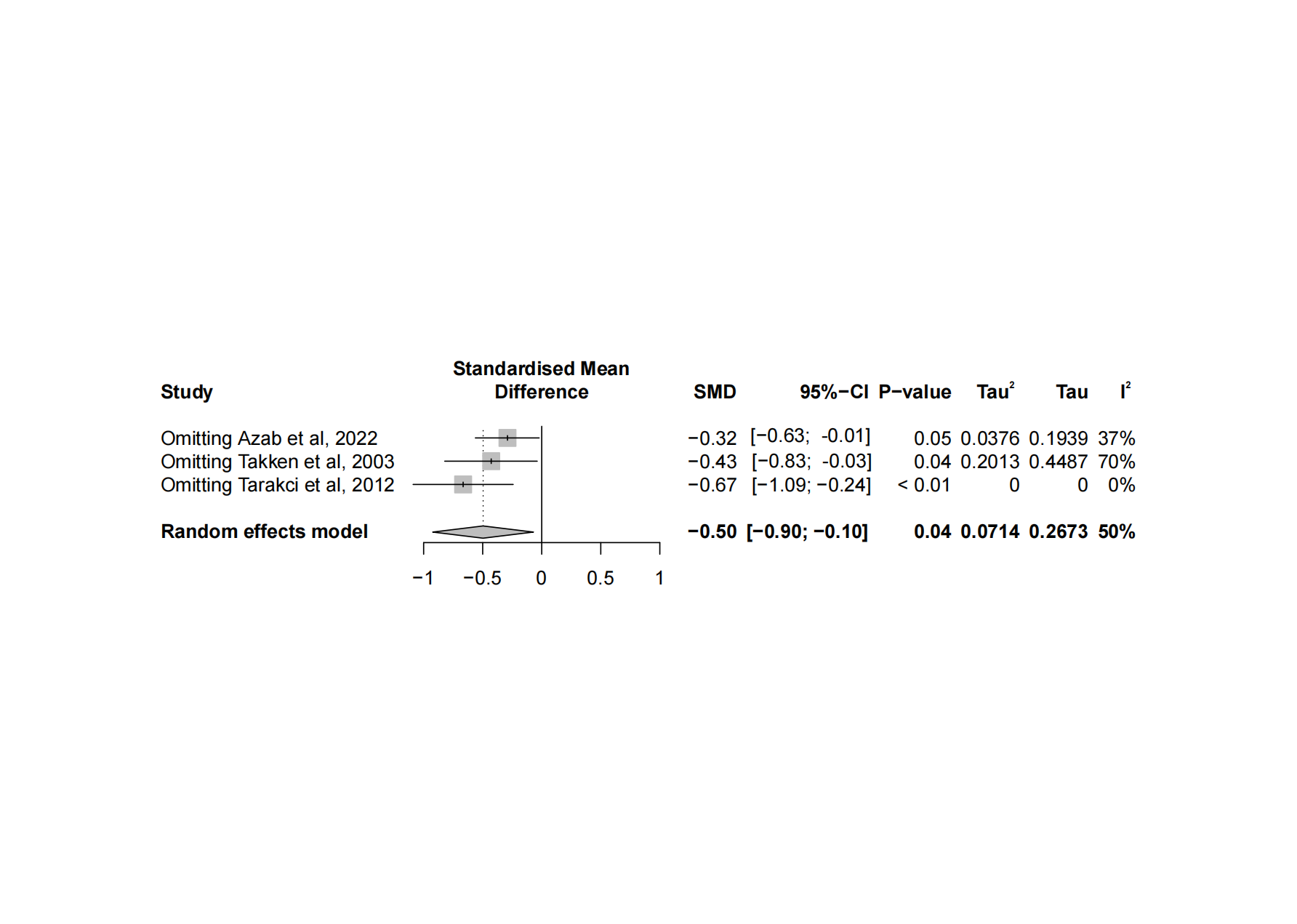


**Figure S13.** Sensitivity analysis of Pain
