# Supplementary material for: Effect of exercise training on heath, quality of life, exercise capacity in juvenile idiopathic arthritis: a meta-analysis of randomized controlled trials
Source: Pediatr Rheumatol Online J. 2024 Mar 4;22:33. doi: 10.1186/s12969-024-00967-3 (PMC10910763; doi:10.1186/s12969-024-00967-3)
Supplement: Supplementary file 4 — Supplementary Material 4 [file 12969_2024_967_MOESM4_ESM.docx]

**Effect of exercise training on heath, quality of life, exercise capacity in juvenile idiopathic arthritis: a meta-analysis of randomized controlled trials**

Wen-yu Liu^ab^, Hui-min Li^c^, Hao Jiang^a^, Wen-kui Zhang^bd^*

**Author Affiliations:**

^a^ Dongshin University, Rojo, South Jeolla Province, South Korea;

^b^ Weifang Institute of Technology, No.9888 Yunmenshan South Road, Qingzhou City, Shandong Province, China;

^c^ Yantai Gold College, No.996 Jinhai Avenue, Binhai New Area, Zhaoyuan, Yantai City, Shandong Province, China;

^d^ Universiti Malaysia Sarawak, Sarawak, East Malaysian Borneo.

**E-mail:**

Wen-yu Liu, lwyv.hi@qq.com;

Hui-min Li, 2650666584@qq.com;

Hao Jiang, chiang0825@163.com;

Wen-kui Zhang, 714599308@qq.com;

***Corresponding Author:**

Wen-kui Zhang,

Weifang Institute of Technology, No.9888 Yunmenshan South Road, Qingzhou City, Shandong Province, China; Universiti Malaysia Sarawak, Sarawak, East Malaysian Borneo (E-mail: 714599308@qq.com).

**Abstract**

**Objective:** Little is known about the efficacy and safety of exercise training on juvenile idiopathic arthritis (JIA). This study aims to investigate the effect of exercise on health, quality of life, and different exercise capacities in individuals with JIA.

**Method:** A comprehensive search of Medline, Embase, Web of Science, and the Cochrane Library was conducted from database inception to October, 2023. Included studies were randomized controlled trials (RCTs) reporting the effects of exercise on JIA patients. Two independent reviewers assessed the literature quality using the Cochrane Collaboration's risk of bias tool. Standardized mean differences (SMD) were combined using random or fixed effects models. The level of evidence was assessed using the Grading of Recommendations Assessment, Development, and Evaluation (GRADE) approach.

**Result:** Five RCTs met the inclusion criteria, containing 216 female participants and 90 males. The meta-analysis results showed that exercise had no significant effect on JIA patients based on the Child Health Assessment Questionnaire (CHAQ) (SMD=-0.32, 95%CI: -0.83, 0.19; I^2^=73.2%, P=0.011) and Quality of Life (QoL) (SMD=0.27, 95%CI: -0.04, 0.58; I^2^=29.4%, P=0.243) and no significant effect on peak oxygen uptake (VO_2_peak). However, exercise significantly reduced visual analog scale (VAS) pain scores in JIA patients (SMD=0.50, 95%CI: -0.90, -0.10; I^2^=50.2%, P=0.134). The quality of evidence assessed by GRADE was moderate to very low.

**Conclusion:** Exercise does not significantly affect the quality of life and exercise capacity in JIA patients but may relieve pain. More RCTs are needed in the future to explore the effects of exercise on JIA.

***Keywords:*** Juvenile idiopathic arthritis; exercise training; quality of life; exercise capacity; meta-analysis

**Introduction**

Juvenile idiopathic arthritis (JIA) is a chronic rheumatic disease of unknown origin common in children, characterized primarily by onset before the age of 16 years and duration of more than 6 weeks ^1,2^. The global incidence is estimated at 1.6 to 23 per 100,000, and the global prevalence is estimated at 3.8 to 400 per 100,000 ^3,4^. JIA presents with symptoms such as arthritic swelling, pain, and stiffness, leading to limited movement. However, it is important to note that each subtype of JIA may exhibit different symptoms ^5,6^. Although some JIA patients have little or no arthritis pain, it is crucial not to overlook the condition, as it can lead to severe complications such as glaucoma, cataracts, and macrophage activation syndrome ^7-9^.

Fortunately, there have been significant advancements in the development of drugs for treating JIA. The typical treatment approach involves the use of nonsteroidal anti-inflammatory drugs (NSAIDs) or intra-articular corticosteroid injections ^2,10^ to conventional anti-rheumatic drugs (csDMARD.) If these do not work, a biological DMARD (bDMARD) is considered the next treatment line ^1^. However, medications usually have several side effects, may be ineffective, and can further exacerbate the disease, leading to JIA joint damage or even irreversible disability ^11^. Exercise is a physical activity that maintains or enhances physical health and is characterized by a variety of forms, a broad audience, few side effects, and high operability, and is widely used in various disease areas such as obesity, cardiovascular disease, chronic obstructive pulmonary disease, diabetes, osteoporosis, cancer, and low back pain ^12-16^. In children with JIA, the muscles around the affected joints are usually wasted, leading to low bone density and, consequently, to osteoporosis in adulthood ^17^. Several studies have shown that exercise, as a "cure-all," is also effective in JIA, helping to build and maintain healthy bone density, muscle strength, and joint mobility, enhance immunity, and improve the quality of life of people with JIA ^18,19^. The above studies overlap in their assertion that exercise may be a more appropriate step-by-step strategy than medication.

The impact of exercise on JIA remains unclear due to conflicting findings in the existing literature. While a meta-analysis suggested that exercise improves the quality of life and exercise capacity in people with JIA ^20^, another study did not yield similar findings ^21^. Moreover, the literature in the former study must be updated, and some new related studies must be included for further analysis ^21^. Although the latter study included relatively more literature, some included trials were non-randomized controlled trials (RCTs), and the trial control group was not uniform, which may lead to excessive heterogeneity in the study and significantly reduce the accuracy of the results ^20^. To address these limitations, our study aims to comprehensively include high-quality RCTs and provide robust research evidence on the effectiveness of exercise on quality of life and exercise capacity in JIA. Our study aims to investigate the effect of exercise on quality of life and exercise capacity in JIA by integrating and analyzing high-quality RCTs. In addition, we systematically described the quality of evidence for the outcomes of interest and their strength of recommendation using the Grading of Recommendations Assessment, Development, and Evaluation (GRADE) method ^22^.

**Methods**

The review was conducted in accordance with the Preferred Reporting Items for Systematic Reviews and Meta-Analyses (PRISMA) guidelines ^23^.

**Search strategies**

A comprehensive search was conducted in Medline, Web of Science, Embase, and Cochrane Library databases from inception until October, 2023. The search was performed using Boolean operators combined with medical subject terms. The searches for relevant studies on the effects of exercise and training on juvenile-onset arthritis were conducted using subject terms such as "child," "adolescent," "juvenile-onset arthritis," "exercise," and "training," as well as free words. Our searches were limited to English language studies. The search strategy is provided on page 2 of the **Appendix page 1.**

**Inclusion and exclusion criteria**

Studies were included in the meta-analysis if they met the following criteria:

1. The participants of the studies were children or adolescents under 19 years of age diagnosed with JIA by a rheumatologist using standard diagnostic methods.

2. Interventions included any type of exercise, such as aerobic, resistance, physical and mental, and multi-form exercise.

3. The control group received no exercise intervention, including usual care, relaxation, and wait-list.

4. The outcomes assessed included health-related outcomes, quality of life-related outcomes, exercise-related outcomes, and pain, measured before and after the intervention.

5. The type of study was randomized controlled trials.

The exclusion criteria were as follows:

1. Studies that included additional types of interventions along with exercise in the intervention group were excluded.

2. Complete data could not be obtained in the study.

3. Review articles or experimental protocols were excluded.

**Study selection**

To ensure that the studies met the inclusion criteria, two authors (Wy.L and Hm.L) conducted independent reviews of all titles, abstracts, and main body sections of the literature. In cases of disagreement between the two authors, a third author (Kw.Z) was consulted.

**Data extraction, outcome measures**

Two independent authors (Wy.L and Hm.L) extracted relevant information from eligible studies, including first author, year of publication, participant demographics (age, sample size), outcomes related to health level, quality of life, exercise capacity, and aerobic level, and information on the control group. If these data were unavailable in the article or additional files, we contacted the corresponding author to obtain them. When two authors disagreed on the information extracted, it was reassessed by a third author (Kw.Z).

Four components were extracted: health level-related outcomes (CHAQ, Child Health Assessment Questionnaire), quality of life-related outcomes (QoL, Quality of Life; PedsQL, Pediatric Quality of Life Inventory), exercise-related outcomes (VO_2_peak, peak oxygen uptake; 6-MWT, 6-min walking test; EPM, Pediatric Escola Paulista de Medicina Range of Motion scale; HRmax, max heart rate), and pain (VAS, visual analog scale; Swollen and tender joints). These results were combined into seven separate categories.

**Quality assessment**

Two authors independently reviewed the included RCTs based on the Cochrane Collaboration's risk-of-bias tool ^24^. Seven risk biases were assessed, including random sequence generation, allocation concealment, participant and personnel blinding, blinding of the outcome assessor, incomplete data, selective reporting, and other sources of bias. These risks were classified as high, unclear, and low.

To assess the quality of evidence for each meta-analysis, the GRADE method was used ^25^. The quality of evidence was determined based on the following entries: risk of bias, inconsistency, indirect evidence, imprecision, and risk of publication bias for each study. The above entries were graded as high, medium, low, and very low quality. High-quality evidence implies that further research is unlikely to substantially alter the confidence in the estimated effect. Very low quality evidence suggests that confidence in the effect estimate is low.

**Statistical analyses**

We conducted a pairwise meta-analysis of studies that met the inclusion criteria. First, Mean and standard deviation (SD) values for the baseline and endpoint measurements were extracted from the included studies. If the required data were unavailable in a study, appropriate methods were used to convert the data into a standard format ^26,27^. Quantitative pooled analyses were conducted using either random-effects or fixed-effects models, depending on the characteristics of the included studies. Given that different scales were used across studies to present experimental results, we calculated standardized mean differences (SMDs) and 95% confidence intervals (CIs), with P<0.05 indicating significance ^24^. The Cochran Q test was used to analyze the presence of heterogeneity in the study, which was high when I^2^>50% or P<0.1 ^28^. The combined SMDs were shown in the forest plot, along with the magnitude of heterogeneity. Contour-enhanced funnel plots were generated to test for study publication bias. In addition, the Egger test was used to check for study bias, with P<0.05 indicating bias ^29,30^. Use a sensitivity analysis method of deletion one by one to examine the heterogeneity sources of the study and test whether different outcomes are stable.

**Results**

**Characteristics of the included studies**

Our initial literature search yielded 495 studies. After excluding duplicate studies (n=128) and irrelevant studies (n=351), 16 studies remained for abstract assessment. 11 studies were excluded for the following reasons: non-RCTs (n=3), control groups that did not meet the inclusion criteria (n=4), studies did not provide data available for analysis (n=3), and mismatch for intervention type (n=1). Finally, five RCTs were included in our meta-analysis, and the screening process is shown in **Figure 1**.

A total of 306 participants were included in the study, primarily women (n=216, 70.6%), with a mean age of 8.7-14.9 years. The duration of the intervention was 12 weeks for four studies and 24 weeks for one. All participants had JIA diagnosed by rheumatologists according to the criteria used: International League of the Associations for Rheumatology or European League of the Associations for Rheumatology. The studies were from America (n=2), Europe (n=2), and Asia (n=1). The outcomes of the four components were grouped into seven categories of indicators for pooled analysis: CHAQ, QoL(PQoL, Pediatric Quality of Life Inventory; Qol, Quality of Life), EPM, VO_2_peak, 6MWT, HRmax, and pain (VAS, Visual Analogue Scale; Swollen and tender joints). Each category may contain a unique test questionnaire scale or measures, or it may contain different ones. In addition, no studies reported adverse events. Detailed demographic characteristics and other information are presented in **Tables 1 and 2**.

**Quality of the included studies**

Of the five studies included, only 1 study was considered to be at low risk of bias in random sequence generation, 1 study was at low risk of bias in allocation concealment, and two studies were at high risk of performance bias. In addition, three studies were at low risk of detection bias. Finally, 1 study was considered at high risk of other bias (**Figure S1, S2**).

According to the GRADE Working Group evidence level, the quality of evidence related to pain was moderate, suggesting a reasonable level of confidence in the effect estimate for pain reduction, indicating that the true effect is likely to be close to the estimated effect, although there is still a possibility of significant differences. The quality of evidence for CHAQ and 6MWT-related outcomes was low, suggesting limited confidence in this effect estimate and that the true effect may differ substantially from the effect estimate. The quality of evidence relating to the quality of life, EPM, VO_2_peak, and HRmax was extremely low, suggesting little confidence in these effect estimates and that the true effect may be significantly different from the effect estimate (**Table 3**).

**Outcomes**

**CHAQ**

Four studies reported the results of CHAQ. Meta-analysis showed no significant improvement in health levels from exercise compared to the control group (SMD=-0.32, 95%CI: -0.83, 0.19). I^2^ (73.2%) and Q (11.19, df=3, P=0.011) statistics highlighted large heterogeneity between studies (**Table 2****, Figure S3**). Due to the limited number of studies (less than 10), it was not feasible to assess publication bias using the funnel plot symmetry test, limiting the possibility of distinguishing between chance and true asymmetry.

**QoL**

A pooled analysis of the three results showed that exercise did not significantly improve quality of life (SMD=0.27, 95%CI: -0.04, 0.58). I^2^ (29.4%) and Q (2.83, df=2, P=0.243) statistics highlighted small heterogeneity between studies (**Table 2, Figure S4**). However, due to the limited number of studies (less than 10), it was not feasible to assess the asymmetry of the funnel plot.

**EPM**

Two studies reported on the outcomes of EPM. Our pooled estimates showed that exercise did not significantly improve exercise capacity compared to the control group (SMD=-0.27, 95CI%: -0.62, 0.09). I^2^ (0.0%) and Q (2.83, df=2, P=0.243) statistics highlighted very low heterogeneity between studies (**Table 2, Figure S5**). Publication bias was not assessed (N<10).

**VO_2_peak**

Three studies reported the results of VO_2_peak. The meta-analysis showed no significant improvement in VO_2_peak levels from exercise compared to the control group (SMD=0.21, 95%CI: -0.10, 0.52). I^2^ (17.5%) and Q (2.4, df=2, P=0.297) statistics highlighted large heterogeneity between studies (**Table 2, Figure S6**). No test of publication bias was performed (N<10).

**6MWT**

Two studies provided data on 6MWT. A pooled analysis of these two studies showed that exercise did not significantly improve 6MWT (SMD=0.29, 95%CI: -0.05, 0.63). I^2^ (0%) and Q (0.11, df=1, P=0.741) statistics highlighted very small heterogeneity between studies (**Table 2, Figure S7**). No test of publication bias was performed (N<10).

**HRmax**

Two studies reported the results of HRmax. Our pooled analysis showed a tendency for the long-term exercise intervention to increase the maximum heart rate of children and adolescents compared to the control group, but it was not statistically significant (SMD=0.77, 95%CI: -0.26, 1.80). I^2^ (77.5%) and Q (4.44, df=1, P=0.035) statistics highlighted very high heterogeneity between studies (**Table 2, Figure S8**). No test of publication bias was performed (N<10).

**Pain**

Pooled analysis of the three outcomes showed that exercise significantly reduced pain levels (SMD=-0.50, 95%CI: -0.90, -0.10). I^2^ (50.2%) and Q (4.02, df=2, P=0.134) statistics highlighted moderate heterogeneity between studies (**Table 2, Figure S9**). No test of publication bias was performed (N<10).

**Sensitivity analysis**

Due to limitations in the included literature, sensitivity analyses were conducted for CHAQ, QoL, VO_2_peak, and pain. After studies were removed one at a time, all outcomes were stable **(Figure S10-13)**. However, in the pain outcome, we found a significant reduction in heterogeneity after removing the Tarakci’s study ^31^, suggesting that this study may be a source of heterogeneity **(Figure S13)**.

**Discussion**

To our knowledge, this study is the first to assess exercise's effect on people with JIA based on high-quality RCTs. We pooled the results of five randomized controlled trials involving 306 individuals for this study to comprehensively assess the effect of exercise on quality of life and exercise capacity in JIA. Interestingly, our findings suggest that exercise did not significantly improve the exercise capacity, health, and quality of life of people with JIA compared to controls. However, exercise significantly improved pain in JIA. According to the GRADE method, the quality of the outcome evidence was very low (very low to moderate), and the results should be interpreted with caution.

**Quality of life and Health status**

Two quality-of-life scales were pooled for this study, including the QoL and PedsQL. The PedsQL is a health-related QoL (HRQoL) scale for rheumatic diseases designed to assess health-related quality of life in children and adolescents with rheumatic diseases such as juvenile idiopathic arthritis, systemic lupus erythematosus, and juvenile-type fibromyalgia, high reliability and validity ^32,33^. Our pooled analysis of QoL and HRQoL revealed that exercise could improve the quality of life in JIA, consistent with the literature ^21^. JIA often presents with unique signs and symptoms such as altered body joint structure, limited range of motion in joints, drowsiness, and fatigue ^1,5^. Lack of exercise and disease-related disability have certain psychosocial implications, which may lead to changes in QoL and HRQoL in JIA ^34,35^. However, exercise can change the body composition of people with JIA, with a decrease in fat mass and an increase in bone and lean mass ^36^, leading to healthy body composition in people with JIA. Exercise also increases self-esteem and self-efficacy and improves sleep quality, thereby increasing QoL in people with JIA ^37^. QoL and HRQoL are important factors associated with personal well-being, and the management of JIA should focus on the potential benefits of exercise. Although there was a trend for exercise to improve quality of life in JIA, the results were not statistically significant, suggesting that the results may change with the inclusion of new studies. Given the limited evidence, it is essential to conduct more high-quality RCTs in the future to confirm the effect of exercise on the quality of life in JIA.

The Child Health Assessment Questionnaire is used to score a child's disability on a scale from 0 to 3, with higher scores representing more severe disability ^38^. One of the main findings of the study was the assessment of CHAQ, and our results suggest that exercise improves the health status of people with JIA. However, contrary to a previous study, the difference was not statistically significant ^20^. Given the limitations of previous research, we place greater confidence in the results of our study. It is now understood that exercise can improve the state of the body in several ways, such as by reducing adiposity, developing the muscular and cardiovascular systems, and improving bone density, balance, and coordination ^39-41^. However, exercise did not significantly change the health status of JIA patients. It is noteworthy that exercise also did not significantly worsen the health status of JIA patients.

**Exercise capacity**

Four outcome indicators, VO_2_peak, 6mwt, EPM, and HRmax, were used to assess exercise capacity in JIA patients, with VO_2_peak, 6mwt, and HRmax showing similar trends and exercise improving these indicators in JIA patients. Interestingly, the magnitude of the benefits of exercise on these indicators was different, with similar benefits for VO_2_peak (SMD: 0.21; 95% CI: -0.10, 0.52) and 6mwt (MD: 0.29; 95% CI: -0.05, 0.62) and the most significant benefit for HRmax (SMD: 0.77; 95% CI: -0.26, 1.80). VO_2_peak has been widely used as an indicator to assess cardiac health ^42^. Usually, the VO_2_peak increases with age and maturity ^43^, and our study demonstrated that exercise significantly increases the benefits of this process through effective heart stimulation in JIA patients. Theoretical indications suggest that improvements in HRmax and VO_2_peak would contribute to an increase in 6mwt, a commonly used measure of exercise capacity. Interestingly, our results indicated that the association between HRmax and 6mwt may be weaker compared to the association between VO_2_peak and 6mwt. However, contrary to expectations, exercise did not lead to a significant improvement in the Exercise Performance Measure (EMP) (SMD: -0.26; 95% CI: -0.62, 0.09) and even showed a negative trend. Exercise is known to improve joint range of motion by building muscle strength and increasing ligament flexibility ^44,45^, and our results contradict conventional knowledge. This discrepancy might be discouraging, but it is essential to consider that exercise could improve specific motor abilities in JIA while potentially having different effects on the overall progression of the condition. Unlike rheumatoid arthritis (RA), exercise improves the ability of joints, such as the joints of the hands, to perform movement^46^. This may be because RA affects not only the joints but also other parts of the body, including the eyes, the skin, the heart, the lungs, the nerves, and the bloodstream^47^. The effect of exercise is superimposed on the various parts of the body, which in turn exhibits an amplification of the effect. In addition, the studies included in our analysis did not show any worsening of JIA, and due to limited original research and the variety of exercise types, we could not characterize the relationship between exercise and JIA progression.

**Pain**

The significant outcome of the study was the improvement in pain among JIA patients, validating the positive effect of exercise in this population, consistent with the literature^20^, which provides the theoretical basis for promoting exercise to this patient population. First, it is well known that physical exercise reduces functional pain by acting on the central mechanisms of pain perception and produces pressure changes at the stress site, which can cause changes in pain perception^48^. Some studies have found that seriously injured soldiers in combat are virtually pain-free, and dancers and athletes who continue to perform strenuous exercise in the face of serious injury feel no pain^49,50^. Second, an increase in pain threshold and tolerance and a decrease in pain rating occur after exercise^51^. A person's pain threshold increased immediately after 40 minutes of running, and cycling continuously for 8 minutes between 200 and 250W loads significantly increased the threshold for dental pain^52,53^. Additional studies have found that exercise alters mechanisms related to the release of adrenocorticotropic hormone and growth hormone, thereby improving symptoms of the disorder and reducing pain^54,55^. However, these possible mechanisms are not specific to JIA and are equally applicable to various chronic diseases. Moreover, we resolved the previous conflicting results regarding the impact of exercise on pain ^21^. Notably, Tarakci's study ^31^ had a higher number of participants drop out, which may affect the credibility of the results. However, adopting the appropriate exercise is undoubtedly associated with a reduction in disease-induced pain ^56^. In the present study, all RCTs included in this meta-analysis had an exercise intervention period of more than twelve weeks. Accordingly, future studies are needed to confirm the effect of short-term exercise on JIA.

**Clinical implications**

This study has several important clinical implications for the management of JIA. First, this study found that although long-term exercise intervention did not significantly improve the overall quality of life and overall health of adolescents with JIA, long-term exercise did not lead to worsening health status in adolescents. This suggests that long-term exercise is safe for JIA. Second, long-term exercise intervention decreases pain levels in patients with JIA, and clinical practitioners should consider prescribing and encouraging exercise interventions for patients with JIA, alongside medication management, taking into account individual circumstances. In summary, regular physiotherapy is essential to JIA management to promote muscle and bone health, optimize body function, and slow disease progression. In the future, it may be necessary to incorporate the natural increase in the exercise capacity of JIA patients themselves, enriching the form of exercise while increasing the intensity to counteract the adverse features of JIA. We also recommend that exercise be an integral part of JIA and other standard care for arthritis.

**Strengths and limitations**

This study possesses several strengths. First, as of May 1, 2023, we included all high-quality RCTs that met strict inclusion and exclusion criteria. Second, we comprehensively included multifaceted outcome indicators, enabling a thorough analysis of the impact of exercise on JIA across multiple levels. Finally, we systematically assessed the quality of evidence for relevant outcomes using the GRADE methodology and provided a strength of recommendation. These strengths enhance the study's rigor, completeness, and high reference value.

In addition, there were several limitations to the study. The most significant one was the small number of RCT trials available. Despite conducting a comprehensive and systematic database search, only 16 controlled trials related to JIA were identified. Out of these, only five trials met the criteria for inclusion due to issues such as the control group being the intervention itself or the intervention group being mixed with other measures, as well as poor experimental design. The limited number of RCTs hindered a more in-depth analysis, and we could not perform regression and subgroup analyses, which could have provided a better understanding of the heterogeneity in the results. Due to the limitations of the included articles, we were unable to assess publication bias using methods such as funnel plots. Lastly, due to limitations in the primary literature, we may not be able to pool other endpoints, such as biomechanical measures for adolescents. This may affect the comprehensiveness of the results.

**Conclusions**

In conclusion, long-term exercise interventions do not significantly improve the quality of life and exercise capacity of JIA patients, but they also do not lead to a deterioration of health status. However, these interventions are effective in relieving pain associated with JIA. Based on our findings, we advocate using appropriate long-term exercise interventions in clinical practice as an adjunct to JIA for pain relief. Furthermore, more randomized controlled trials are needed to investigate the optimal exercise interventions for JIA and determine the dose that achieves the minimum clinically important difference.

List of abbreviations

| CHAQ | Child Health Assessment Questionnaire |
| --- | --- |
| 6MWT | 6-min walking test |
| EPM | Pediatric Escola Paulista de Medicina Range of Motion scale |
| HRmax | Max heart rate |
| JIA | Juvenile idiopathic arthritis |
| QoL | Quality of Life |
| RA | Rheumatoid arthritis |
| RCT | Randomized controlled trial |
| VAS | Visual analog scale |

**Declarations**

**Ethics approval and consent to participate**

Not applicable.

**Consent for publication**

Not applicable.

**Availability of data and material**

Since all analyses were performed based on previously published studies, no ethical approval or patient consent was required.

**Competing interests**

All authors declare that they have no competing interests.

**Funding**

Not applicable.

**Authors' contributions**

W-Y L served as principal author and had full access to all the data in the study, takes responsibility for the accuracy of the data analysis, and the integrity of the data. H-M L and HJ contributed to the conception and design. W-Y L, H-M L and HJ contributed to data acquisition and interpretation. W-Y L and HJ contributed to draft of the manuscript. W-K Z contributed to revise of the article and final approval.

**Acknowledgements**

The authors are grateful for the support provided by Dongshin University.

**Reference**

1 Martini, A. *et al.* Juvenile idiopathic arthritis. *Nature reviews. Disease primers* **8**, 5, doi:10.1038/s41572-021-00332-8 (2022).

2 Prakken, B., Albani, S. & Martini, A. Juvenile idiopathic arthritis. *Lancet* **377**, 2138-2149, doi:10.1016/s0140-6736(11)60244-4 (2011).

3 Palman, J., Shoop-Worrall, S., Hyrich, K. & McDonagh, J. E. Update on the epidemiology, risk factors and disease outcomes of Juvenile idiopathic arthritis. *Best practice & research. Clinical rheumatology* **32**, 206-222, doi:10.1016/j.berh.2018.10.004 (2018).

4 Thierry, S., Fautrel, B., Lemelle, I. & Guillemin, F. Prevalence and incidence of juvenile idiopathic arthritis: a systematic review. *Joint Bone Spine* **81**, 112-117, doi:10.1016/j.jbspin.2013.09.003 (2014).

5 Hemke, R. *et al.* Frequency of joint involvement in juvenile idiopathic arthritis during a 5-year follow-up of newly diagnosed patients: implications for MR imaging as outcome measure. *Rheumatol Int* **35**, 351-357, doi:10.1007/s00296-014-3108-x (2015).

6 Hansmann, S., Benseler, S. M. & Kuemmerle-Deschner, J. B. Dynamic knee joint function in children with juvenile idiopathic arthritis (JIA). *Pediatric rheumatology online journal* **13**, 8, doi:10.1186/s12969-015-0004-1 (2015).

7 Sen, E. S., Dick, A. D. & Ramanan, A. V. Uveitis associated with juvenile idiopathic arthritis. *Nature reviews. Rheumatology* **11**, 338-348, doi:10.1038/nrrheum.2015.20 (2015).

8 Foster, H., Rapley, T. & May, C. Juvenile idiopathic arthritis: improved outcome requires improved access to care. *Rheumatology (Oxford, England)* **49**, 401-403, doi:10.1093/rheumatology/kep347 (2010).

9 Cimaz, R. Systemic-onset juvenile idiopathic arthritis. *Autoimmunity reviews* **15**, 931-934, doi:10.1016/j.autrev.2016.07.004 (2016).

10 Ravelli, A. & Martini, A. Juvenile idiopathic arthritis. *Lancet* **369**, 767-778, doi:10.1016/s0140-6736(07)60363-8 (2007).

11 Giancane, G. *et al.* Disease activity and damage in juvenile idiopathic arthritis: methotrexate era versus biologic era. *Arthritis Res Ther* **21**, 168, doi:10.1186/s13075-019-1950-7 (2019).

12 Barker, K. & Eickmeyer, S. Therapeutic Exercise. *The Medical clinics of North America* **104**, 189-198, doi:10.1016/j.mcna.2019.10.003 (2020).

13 Neil-Sztramko, S. E., Caldwell, H. & Dobbins, M. School-based physical activity programs for promoting physical activity and fitness in children and adolescents aged 6 to 18. *The Cochrane database of systematic reviews* **9**, Cd007651, doi:10.1002/14651858.CD007651.pub3 (2021).

14 Owen, P. J. *et al.* Which specific modes of exercise training are most effective for treating low back pain? Network meta-analysis. *Br J Sports Med* **54**, 1279-1287, doi:10.1136/bjsports-2019-100886 (2020).

15 Chen, H. *et al.* Exercise training maintains cardiovascular health: signaling pathways involved and potential therapeutics. *Signal transduction and targeted therapy* **7**, 306, doi:10.1038/s41392-022-01153-1 (2022).

16 Hou, L. *et al.* Exercise modalities for type 2 diabetes: A systematic review and network meta-analysis of randomized trials. *Diabetes/metabolism research and reviews* **39**, e3591, doi:10.1002/dmrr.3591 (2023).

17 Stagi, S. *et al.* Bone mass and quality in patients with juvenile idiopathic arthritis: longitudinal evaluation of bone-mass determinants by using dual-energy x-ray absorptiometry, peripheral quantitative computed tomography, and quantitative ultrasonography. *Arthritis Res Ther* **16**, R83, doi:10.1186/ar4525 (2014).

18 Long, A. R. & Rouster-Stevens, K. A. The role of exercise therapy in the management of juvenile idiopathic arthritis. *Current opinion in rheumatology* **22**, 213-217, doi:10.1097/BOR.0b013e328335d1a2 (2010).

19 Rochette, E., Saidi, O., Merlin, É. & Duché, P. Physical activity as a promising alternative for young people with juvenile idiopathic arthritis: Towards an evidence-based prescription. *Front Immunol* **14**, 1119930, doi:10.3389/fimmu.2023.1119930 (2023).

20 Kuntze, G. *et al.* Exercise Therapy in Juvenile Idiopathic Arthritis: A Systematic Review and Meta-Analysis. *Arch Phys Med Rehabil* **99**, 178-193.e171, doi:10.1016/j.apmr.2017.05.030 (2018).

21 Takken, T. *et al.* Exercise therapy in juvenile idiopathic arthritis. *The Cochrane database of systematic reviews* **2008**, Cd005954, doi:10.1002/14651858.CD005954.pub2 (2008).

22 Guyatt, G. H. *et al.* GRADE: an emerging consensus on rating quality of evidence and strength of recommendations. *Bmj* **336**, 924-926, doi:10.1136/bmj.39489.470347.AD (2008).

23 Page, M. J. *et al.* The PRISMA 2020 statement: an updated guideline for reporting systematic reviews. *BMJ (Clinical research ed.)* **372**, n71, doi:10.1136/bmj.n71 (2021).

24 JP, H. *Cochrane Handbook for Systematic Reviews of Interventions Version 5.0. 2*, <<https://training.cochrane.org/handbook/archive/v5.0.2/>> (2009).

25 Atkins, D. *et al.* Grading quality of evidence and strength of recommendations. *BMJ (Clinical research ed.)* **328**, 1490, doi:10.1136/bmj.328.7454.1490 (2004).

26 Hedges, L. V. Distribution Theory for Glass's Estimator of Effect size and Related Estimators. *Journal of Educational Statistics* **6**, 107-128, doi:10.3102/10769986006002107 (1981).

27 Wan, X., Wang, W., Liu, J. & Tong, T. Estimating the sample mean and standard deviation from the sample size, median, range and/or interquartile range. *BMC Medical Research Methodology* **14**, 135, doi:10.1186/1471-2288-14-135 (2014).

28 Higgins, J. P. & Thompson, S. G. Quantifying heterogeneity in a meta-analysis. *Statistics in medicine* **21**, 1539-1558, doi:10.1002/sim.1186 (2002).

29 Egger, M., Juni, P., Bartlett, C., Holenstein, F. & Sterne, J. How important are comprehensive literature searches and the assessment of trial quality in systematic reviews? Empirical study. *Health technology assessment (Winchester, England)* **7**, 1-76 (2003).

30 Sterne, J. A. *et al.* Recommendations for examining and interpreting funnel plot asymmetry in meta-analyses of randomised controlled trials. *BMJ (Clinical research ed.)* **343** (2011).

31 Tarakci, E., Yeldan, I., Baydogan, S. N., Olgar, S. & Kasapcopur, O. Efficacy of a land-based home exercise programme for patients with juvenile idiopathic arthritis: a randomized, controlled, single-blind study. *J Rehabil Med* **44**, 962-967, doi:10.2340/16501977-1051 (2012).

32 Flowers, S. R. & Kashikar-Zuck, S. Measures of juvenile fibromyalgia: Functional Disability Inventory (FDI), Modified Fibromyalgia Impact Questionnaire-Child Version (MFIQ-C), and Pediatric Quality of Life Inventory (PedsQL) 3.0 Rheumatology Module Pain and Hurt Scale. *Arthritis Care Res (Hoboken)* **63 Suppl 11**, S431-437, doi:10.1002/acr.20639 (2011).

33 Varni, J. W., Burwinkle, T. M. & Szer, I. S. The PedsQL Multidimensional Fatigue Scale in pediatric rheumatology: reliability and validity. *The Journal of rheumatology* **31**, 2494-2500 (2004).

34 Müller-Godeffroy, E., Lehmann, H., Küster, R. M. & Thyen, U. [Quality of life and psychosocial adaptation in children and adolescents with juvenile idiopathic arthritis and reactive arthritis]. *Zeitschrift fur Rheumatologie* **64**, 177-187, doi:10.1007/s00393-005-0652-5 (2005).

35 Amine, B., Rostom, S., Benbouazza, K., Abouqal, R. & Hajjaj-Hassouni, N. Health related quality of life survey about children and adolescents with juvenile idiopathic arthritis. *Rheumatol Int* **29**, 275-279, doi:10.1007/s00296-008-0672-y (2009).

36 Stoner, L. *et al.* Efficacy of Exercise Intervention for Weight Loss in Overweight and Obese Adolescents: Meta-Analysis and Implications. *Sports medicine (Auckland, N.Z.)* **46**, 1737-1751, doi:10.1007/s40279-016-0537-6 (2016).

37 Axelsdóttir, B., Biedilae, S., Sagatun, Å., Nordheim, L. V. & Larun, L. Review: Exercise for depression in children and adolescents - a systematic review and meta-analysis. *Child and adolescent mental health* **26**, 347-356, doi:10.1111/camh.12438 (2021).

38 Lam, C., Young, N., Marwaha, J., McLimont, M. & Feldman, B. M. Revised versions of the Childhood Health Assessment Questionnaire (CHAQ) are more sensitive and suffer less from a ceiling effect. *Arthritis Rheum* **51**, 881-889, doi:10.1002/art.20820 (2004).

39 Low, D. C., Walsh, G. S. & Arkesteijn, M. Effectiveness of Exercise Interventions to Improve Postural Control in Older Adults: A Systematic Review and Meta-Analyses of Centre of Pressure Measurements. *Sports medicine (Auckland, N.Z.)* **47**, 101-112, doi:10.1007/s40279-016-0559-0 (2017).

40 Mages, M. *et al.* Exercise Effects on Bone Mineral Density in Men. *Nutrients* **13**, doi:10.3390/nu13124244 (2021).

41 Fiuza-Luces, C. *et al.* Exercise benefits in cardiovascular disease: beyond attenuation of traditional risk factors. *Nature reviews. Cardiology* **15**, 731-743, doi:10.1038/s41569-018-0065-1 (2018).

42 Ross, R. *et al.* Importance of Assessing Cardiorespiratory Fitness in Clinical Practice: A Case for Fitness as a Clinical Vital Sign: A Scientific Statement From the American Heart Association. *Circulation* **134**, e653-e699, doi:10.1161/cir.0000000000000461 (2016).

43 Armstrong, N., Tomkinson, G. & Ekelund, U. Aerobic fitness and its relationship to sport, exercise training and habitual physical activity during youth. *Br J Sports Med* **45**, 849-858, doi:10.1136/bjsports-2011-090200 (2011).

44 Alizadeh, S. *et al.* Resistance Training Induces Improvements in Range of Motion: A Systematic Review and Meta-Analysis. *Sports medicine (Auckland, N.Z.)* **53**, 707-722, doi:10.1007/s40279-022-01804-x (2023).

45 Sweeney, F. C. *et al.* Aerobic and Resistance Exercise Improves Shoulder Function in Women Who Are Overweight or Obese and Have Breast Cancer: A Randomized Controlled Trial. *Physical therapy* **99**, 1334-1345, doi:10.1093/ptj/pzz096 (2019).

46 Hammond, A. & Prior, Y. The effectiveness of home hand exercise programmes in rheumatoid arthritis: a systematic review. *British medical bulletin* **119**, 49-62, doi:10.1093/bmb/ldw024 (2016).

47 Majithia, V. & Geraci, S. A. Rheumatoid arthritis: diagnosis and management. *The American journal of medicine* **120**, 936-939, doi:10.1016/j.amjmed.2007.04.005 (2007).

48 Koltyn, K. F. Analgesia following exercise: a review. *Sports medicine (Auckland, N.Z.)* **29**, 85-98, doi:10.2165/00007256-200029020-00002 (2000).

49 Heecher, H. K. Pain in men wounded in battle. *Ann Surg* **123**, 96-105 (1946).

50 Beecher, H. K. Relationship of significance of wound to pain experienced. *J Am Med Assoc* **161**, 1609-1613, doi:10.1001/jama.1956.02970170005002 (1956).

51 Droste, C., Greenlee, M. W., Schreck, M. & Roskamm, H. Experimental pain thresholds and plasma beta-endorphin levels during exercise. *Med Sci Sports Exerc* **23**, 334-342 (1991).

52 Black, J., Chesher, G. B., Starmer, G. A. & Egger, G. The painlessness of the long distance runner. *The Medical journal of Australia* **1**, 522-523 (1979).

53 Kemppainen, P., Pertovaara, A., Huopaniemi, T., Johansson, G. & Karonen, S. L. Modification of dental pain and cutaneous thermal sensitivity by physical exercise in man. *Brain research* **360**, 33-40, doi:10.1016/0006-8993(85)91217-x (1985).

54 Kemppainen, P., Pertovaara, A., Huopaniemi, T. & Johansson, G. Elevation of dental pain threshold induced in man by physical exercise is not reversed by cyproheptadine-mediated suppression of growth hormone release. *Neurosci Lett* **70**, 388-392, doi:10.1016/0304-3940(86)90585-9 (1986).

55 Kemppainen, P., Paalasmaa, P., Pertovaara, A., Alila, A. & Johansson, G. Dexamethasone attenuates exercise-induced dental analgesia in man. *Brain research* **519**, 329-332, doi:10.1016/0006-8993(90)90096-t (1990).

56 Feldman, D. E. *et al.* Effects of adherence to treatment on short-term outcomes in children with juvenile idiopathic arthritis. *Arthritis Rheum* **57**, 905-912, doi:10.1002/art.22907 (2007).
