# Supplementary material for: Effect of exercise training on heath, quality of life, exercise capacity in juvenile idiopathic arthritis: a meta-analysis of randomized controlled trials
Source: Pediatr Rheumatol Online J. 2024 Mar 4;22:33. doi: 10.1186/s12969-024-00967-3 (PMC10910763; doi:10.1186/s12969-024-00967-3)
Supplement: Supplementary file 3 — Supplementary Material 3 [file 12969_2024_967_MOESM3_ESM.docx]

3th January, 2024

Professors

Editor-in-chief

**Pediatric Rheumatology**

Manuscript ID: **PROJ-D-23-00212**

Manuscript Title: **Effect of exercise training on heath, quality of life, exercise capacity, in juvenile idiopathic arthritis: a meta-analysis of randomized controlled trials**

Dear Editor,

Thank you for your letter in 2th January, 2024 on behalf of my co-authors, we thank you very much for giving us an opportunity to revise our manuscript, we appreciate editor and reviewers very much for their positive and constructive comments and suggestions on our manuscript.

Appended to this letter is our point-by-point response to the comments raised by the reviewer. The comments are reproduced and our responses are given directly afterward in a different color (highlight yellow).

We would like to express our great appreciation to you and reviewers for comments on our paper. We hope that we have responded satisfactorily to the constructive comments of the reviewers, and the paper will now be suitable for publication in the **Pediatric Rheumatology**.

Best wishes,

Wen-kui Zhang

Thank you for your letter and for the reviewers’ comments concerning our manuscript entitled “**Effect of exercise training on heath, quality of life, exercise capacity, in juvenile idiopathic arthritis: a meta-analysis of randomized controlled trials (Manuscript ID: PROJ-D-23-00212).** Those comments are all valuable and very helpful for revising and improving our paper, as well as the important guiding significance to our researches. We have studied comments carefully and have made correction which we hope meet with approval. Revised portion are marked in highlight yellow (normal revision) in the paper. The main corrections in the paper and the responds to the reviewer’s comments are as flowing:

**Comments from the Reviewer 1:**

1. **While the study provides valuable insights, there are several scientific flaws in the methodology and results that need to be considered and make the conclusions regarding exercise in JIA patients. unreliable.**

**Response: Thank you for underlining this deficiency. This study was conducted strictly per the Preferred Reporting Items for Systematic Reviews and Meta-Analyses (PRISMA) guidelines [1], and the included studies were randomized controlled trials with a high degree of confidence. Based on that, we consider our findings to be reliable (e.g., add source of heterogeneity analysis for outcomes with high heterogeneity). In addition, we will address and respond to some of the methodological and outcome flaws you have pointed out below. We believe that with your and our joint efforts, the quality of this study can be further improved to provide a reference for juvenile idiopathic arthritis.**

**Reference:**

**[1] Page MJ, McKenzie JE, Bossuyt PM, Boutron I, Hoffmann TC, Mulrow CD, Shamseer L, Tetzlaff JM, Akl EA, Brennan SE, Chou R, Glanville J, Grimshaw JM, Hróbjartsson A, Lalu MM, Li T, Loder EW, Mayo-Wilson E, McDonald S, McGuinness LA, Stewart LA, Thomas J, Tricco AC, Welch VA, Whiting P, Moher D. The PRISMA 2020 statement: an updated guideline for reporting systematic reviews. BMJ. 2021 Mar 29;372:n71. doi: 10.1136/bmj.n71. PMID: 33782057; PMCID: PMC8005924.**

2. **-Limited Number of Studies and Participants: The analysis only includes 5 RCTs (total 306 participants) which may limit the generalizability of the findings and may not provide a comprehensive representation of the population with JIA.**

**Response: Thank you for this comment. Since there is less original literature in the area of exercise interventions for juvenile arthritis (the initial search yielded only 495 records), there are fewer studies to include in a meta-analysis, and we have to recognize this limitation. However, based on the specificity of this study, we were more concerned about its quality. With a precise search strategy and strict inclusion and exclusion criteria, we could better analyze the effects of exercise on various aspects of juvenile arthritis. In the future, we may need more large randomized controlled trials to study the efficacy of exercise. In addition, in previous meta-analyses, there were also cases where there were fewer included literature, but they all had good reliability [1, 2].**

**Reference:**

[1] **Neuen BL, Young T, Heerspink HJL, Neal B, Perkovic V, Billot L, Mahaffey KW, Charytan DM, Wheeler DC, Arnott C, Bompoint S, Levin A, Jardine MJ. SGLT2 inhibitors for the prevention of kidney failure in patients with type 2 diabetes: a systematic review and meta-analysis. Lancet Diabetes Endocrinol. 2019 Nov;7(11):845-854. doi: 10.1016/S2213-8587(19)30256-6. Epub 2019 Sep 5. Erratum in: Lancet Diabetes Endocrinol. 2019 Dec;7(12):e23. PMID: 31495651.**
[2] **Valle JW, Furuse J, Jitlal M, Beare S, Mizuno N, Wasan H, Bridgewater J, Okusaka T. Cisplatin and gemcitabine for advanced biliary tract cancer: a meta-analysis of two randomised trials. Ann Oncol. 2014 Feb;25(2):391-8. doi: 10.1093/annonc/mdt540. Epub 2013 Dec 18. PMID: 24351397.**
3. **- The GRADE assessment indicates moderate to very low quality of evidence. This raises concerns about the reliability of the findings and the conclusions drawn.**

**Response: Thank you for this comment. We recognize that a moderate to low levels in GRADE may result in less reliable studies. However, it is normal for GRADE to be moderately low: We strictly followed the assessment principles of grade and objectively evaluated the included literature, not a subjective judgment. Due to the risk of bias, inconsistency, and indirectness, we had to downgrade the rating. We believe that while conducting a rigorous GRADE impacts the study's reliability, this impact is relatively small. Moreover, GRADE increases overall rigor and scientific validity [1]. Lower GRADE levels were also present in previous meta-analyses related to exercise interventions [2, 3] but had less impact on the reliability of the studies.**

**Reference:**

[1] **Atkins D, Best D, Briss PA, Eccles M, Falck-Ytter Y, Flottorp S, Guyatt GH, Harbour RT, Haugh MC, Henry D, Hill S, Jaeschke R, Leng G, Liberati A, Magrini N, Mason J, Middleton P, Mrukowicz J, O'Connell D, Oxman AD, Phillips B, Schünemann HJ, Edejer T, Varonen H, Vist GE, Williams JW Jr, Zaza S; GRADE Working Group. Grading quality of evidence and strength of recommendations. BMJ. 2004 Jun 19;328(7454):1490. doi: 10.1136/bmj.328.7454.1490. PMID: 15205295; PMCID: PMC428525.**
[2] **Martínez-Calderon J, Casuso-Holgado MJ, Muñoz-Fernandez MJ, Garcia-Muñoz C, Heredia-Rizo AM. Yoga-based interventions may reduce anxiety symptoms in anxiety disorders and depression symptoms in depressive disorders: a systematic review with meta-analysis and meta-regression. Br J Sports Med. 2023 Nov;57(22):1442-1449. doi: 10.1136/bjsports-2022-106497. Epub 2023 Jun 27. PMID: 37369553.**
[3] **de Zoete RM, Armfield NR, McAuley JH, Chen K, Sterling M. Comparative effectiveness of physical exercise interventions for chronic non-specific neck pain: a systematic review with network meta-analysis of 40 randomised controlled trials. Br J Sports Med. 2020 Nov 2:bjsports-2020-102664. doi: 10.1136/bjsports-2020-102664. Epub ahead of print. PMID: 33139256.**
4. **-Heterogeneity: The I^2^ values for CHAQ and VAS indicate moderate to high heterogeneity among the included studies. This raises concerns about the consistency of the results and indicates that the studies may differ significantly in terms of populations, interventions, or outcomes. Suggest exploring the potential sources of heterogeneity.**

**Response: Thank you for underlining this deficiency. Following your suggestion, we explored the sources of heterogeneity in these two results. Due to the small amount of literature included, we explored sources of heterogeneity using a deletion one by one approach. After analysis, we did not find a source of heterogeneity for the outcome CHAQ (Figure 1). However, in the pain outcome, we found a significant reduction in heterogeneity (I^2^=0) after removing the Tarakci et al [1] study, suggesting that this study may be a source of heterogeneity (Figure 2). In addition, we made corresponding changes in the Methods, Results, and Supplementary file sections. In addition, we made corresponding changes in the Methods, Results, and Supplementary file sections. (Page 7, line 180-182; Page 10, line 276-280) (Supplementary appendix 4: Sensitivity analysis)**

**Figure 1. Sensitivity analysis of CHAQ**

**Figure 2. Sensitivity analysis of Pain**

**Reference:**

[1] **Tarakci E, Yeldan I, Baydogan SN, Olgar S, Kasapcopur O. Efficacy of a land-based home exercise programme for patients with juvenile idiopathic arthritis: a randomized, controlled, single-blind study. J Rehabil Med. 2012 Nov;44(11):962-7. doi: 10.2340/16501977-1051. PMID: 23027068.**
5. **-Other outcome measures such as disease activity scores, biomechanical measures might be helpful tools to assess the effect of exercise on disease outcomes as the outcomes used in the above studies may not fully capture the diverse effects of exercise on JIA. Thus, the statement on the effect of exercise on JIA patients might not be fully reliable.**

**Response: Thank you for this comment. Previous studies reported that pain is the main symptom of JIA [1]; our meta-analysis pooled the outcomes about health level-related outcomes (CHAQ, Child Health Assessment Questionnaire), quality of life-related outcomes (QoL, Quality of Life; PedsQL, Pediatric Quality of Life Inventory), exercise-related outcomes (VO_2_peak, peak oxygen uptake; 6-MWT, 6-min walking test; and EPM, Pediatric Escola Paulista de Medicina Range of Motion scale; HRmax, max heart rate). in addition to the meta-analysis about pain, which responded to the effect of exercise interventions on the aspect of JIA. In addition, we believe that EPM also responds to the level of disease activity to some extent. However, due to the limitations of the original literature, we may not be able to pool other outcomes, such as biomechanical measures. We will elaborate on this point in Limitations. (Page 14, line 391-396)**

**Reference:**

[1] **Kimura Y, Walco GA. Treatment of chronic pain in pediatric rheumatic disease. Nat Clin Pract Rheumatol. 2007 Apr;3(4):210-8. doi: 10.1038/ncprheum0458. PMID: 17396106.**

**Comments from the Reviewer 2:**

1. **The article is interesting in methods and lots of parameters have been analyzed. I suggest to add more pathophysiological information in the discussion about the way physical exercise can relieve pain in chronic diseases, since this is the main findings of the paper. It is known that physical exercise can reduce also functional pain by acting on central mechanisms of pain perception; it also works in somatic symptoms disorders and this mechanism is not specific to JIA but to several chronic diseases.**

**Response: Thank you for pointing this important suggestion. Following your suggestion, we have added more information about the physiology of the cases to our discussion, and we have also added the examples of pain perception and somatic symptom disorders that you suggested. This is very important. See below for detailed modifications:**

**First, it is well known that physical exercise reduces functional pain by acting on the central mechanisms of pain perception and produces pressure changes at the stress site, which can cause changes in pain perception [1]. Some studies have found that seriously injured soldiers in combat are virtually pain-free, and dancers and athletes who continue to perform strenuous exercise in the face of serious injury feel no pain [2, 3]. Second, an increase in pain threshold and tolerance and a decrease in pain rating occur after exercise [4]. A person's pain threshold increased immediately after 40 minutes of running, and cycling continuously for 8 minutes between 200 and 250W loads significantly increased the threshold for dental pain [5, 6]. Additional studies have found that exercise alters mechanisms related to the release of adrenocorticotropic hormone and growth hormone, thereby improving symptoms of the disorder and reducing pain [7, 8]. However, these possible mechanisms are not specific to JIA and are equally applicable to various chronic diseases. (Page 13-14, line 361-373)**

**Reference:**

[1] **Koltyn KF. Analgesia following exercise: a review. Sports Med. 2000 Feb;29(2):85-98. doi: 10.2165/00007256-200029020-00002. PMID: 10701712.**
[2] **HEECHER HK. Pain in men wounded in battle. Ann Surg. 1946 Jan;123:96-105. PMID: 21009341.**
[3] **BEECHER HK. Relationship of significance of wound to pain experienced. J Am Med Assoc. 1956 Aug 25;161(17):1609-13. doi: 10.1001/jama.1956.02970170005002. PMID: 13345630.**
[4] **Droste C, Greenlee MW, Schreck M, Roskamm H. Experimental pain thresholds and plasma beta-endorphin levels during exercise. Med Sci Sports Exerc. 1991 Mar;23(3):334-42. PMID: 2020272.**
[5] **Black J, Chesher GB, Starmer GA, Egger G. The painlessness of the long distance runner. Med J Aust. 1979 Jun 2;1(11):522-3. PMID: 470706.**
[6] **Kemppainen P, Pertovaara A, Huopaniemi T, Johansson G, Karonen SL. Modification of dental pain and cutaneous thermal sensitivity by physical exercise in man. Brain Res. 1985 Dec 23;360(1-2):33-40. doi: 10.1016/0006-8993(85)91217-x. PMID: 3000534.**
[7] **Kemppainen P, Pertovaara A, Huopaniemi T, Johansson G. Elevation of dental pain threshold induced in man by physical exercise is not reversed by cyproheptadine-mediated suppression of growth hormone release. Neurosci Lett. 1986 Oct 20;70(3):388-92. doi: 10.1016/0304-3940(86)90585-9. PMID: 3774237.**
[8] **Kemppainen P, Paalasmaa P, Pertovaara A, Alila A, Johansson G. Dexamethasone attenuates exercise-induced dental analgesia in man. Brain Res. 1990 Jun 11;519(1-2):329-32. doi: 10.1016/0006-8993(90)90096-t. PMID: 2168784.**
2. **Also, I suggest to include some words on the role of physical therapy in such patients and possible new strategies in this field.**

**Response: Thank you for the suggestion. Following up on your comments, we have made some observations about the role of physiotherapy in JIA patients and possible new strategies in this field. See below for detailed modifications:**

**In summary, regular physiotherapy is essential to JIA management to promote muscle and bone health, optimize body function, and slow disease progression. In the future, it may be necessary to incorporate the natural increase in the exercise capacity of JIA patients themselves, enriching the form of exercise while increasing the intensity to counteract the adverse features of JIA. We also recommend that exercise be an integral part of JIA and other standard care for arthritis. (Page 14, line 391-396)**

**Comments from the Reviewer 3:**

**Dear Authors, I congratulate you on your works. Even if there are several limitations, the messages reported are important**

**Response: Thank you for your comments and we will continue to improve the quality of our research by making changes based on the comments of other reviewers. In the future, we will do deeper research around the limitations in the article.**

**Comments from the Reviewer 4:**

1. **The objective was clear and well-defined: to investigate the effect of exercise on health, quality of life, and exercise capacities in individuals with juvenile idiopathic arthritis (JIA). The authors clearly outline the strengths of the study, such as inclusion of high-quality RCTs and comprehensive outcome indicators, while acknowledging limitations, including the small number of available RCTs.**

**Response: Thank you for your comments. Your systematic summary of our manuscript is a great encouragement to us, and we couldn't agree more with what you said. Once again, thank you for your comments.**

2. **In the case of a paucity of available RCTs, what information is available for adults with rheumatoid arthritis and other conditions should be discussed in the discussion. Whether the results are consistent with RA in adults or, on the other hand, different from those in adults and characteristic of JIA in children is an important point for future research, and we believe that the information will be clearer if discussed.**

**Response: Thank you for pointing this important suggestion. Following your suggestion, we explored some available information on JIA and other arthritis in our discussion. JIA and other arthritis are distinguished by some differences in that they have some of the characteristics of childhood JIA and other body parts that are affected, such as the eyes, skin, heart, lungs, nerves, and blood. Meanwhile, we give some suggestions. See below for detailed modifications:**

**Modifications 1: Unlike rheumatoid arthritis (RA), exercise improves the ability of joints, such as the joints of the hands, to perform movement [1]. This may be because RA affects not only the joints but also other parts of the body, including the eyes, the skin, the heart, the lungs, the nerves, and the bloodstream [2]. The effect of exercise is superimposed on the various parts of the body, which in turn exhibits an amplification of the effect. (Page 13, line 347-352)**

**Modifications 2: First, it is well known that physical exercise reduces functional pain by acting on the central mechanisms of pain perception and produces pressure changes at the stress site, which can cause changes in pain perception [3]. Some studies have found that seriously injured soldiers in combat are virtually pain-free, and dancers and athletes who continue to perform strenuous exercise in the face of serious injury feel no pain [4, 5]. Second, an increase in pain threshold and tolerance and a decrease in pain rating occur after exercise [6]. A person's pain threshold increased immediately after 40 minutes of running, and cycling continuously for 8 minutes between 200 and 250W loads significantly increased the threshold for dental pain [7, 8]. Additional studies have found that exercise alters mechanisms related to the release of adrenocorticotropic hormone and growth hormone, thereby improving symptoms of the disorder and reducing pain [9, 10]. However, these possible mechanisms are not specific to JIA and are equally applicable to various chronic diseases. (Page 13-14, line 361-373)**

**Modifications 3: In summary, regular physiotherapy is essential to JIA management to promote muscle and bone health, optimize body function, and slow disease progression. In the future, it may be necessary to incorporate the natural increase in the exercise capacity of JIA patients themselves, enriching the form of exercise while increasing the intensity to counteract the adverse features of JIA. We also recommend that exercise be an integral part of JIA and other standard care for arthritis. (Page 14, line 391-396)**

**Reference:**

[1] **Hammond A, Prior Y. The effectiveness of home hand exercise programmes in rheumatoid arthritis: a systematic review. Br Med Bull. 2016 Sep;119(1):49-62. doi: 10.1093/bmb/ldw024. Epub 2016 Jun 30. PMID: 27365455.**
[2] **Majithia V, Geraci SA. Rheumatoid arthritis: diagnosis and management. Am J Med. 2007 Nov;120(11):936-9. doi: 10.1016/j.amjmed.2007.04.005. PMID: 17976416.**
[3] **Koltyn KF. Analgesia following exercise: a review. Sports Med. 2000 Feb;29(2):85-98. doi: 10.2165/00007256-200029020-00002. PMID: 10701712.**
[4] **HEECHER HK. Pain in men wounded in battle. Ann Surg. 1946 Jan;123:96-105. PMID: 21009341.**
[5] **BEECHER HK. Relationship of significance of wound to pain experienced. J Am Med Assoc. 1956 Aug 25;161(17):1609-13. doi: 10.1001/jama.1956.02970170005002. PMID: 13345630.**
[6] **Droste C, Greenlee MW, Schreck M, Roskamm H. Experimental pain thresholds and plasma beta-endorphin levels during exercise. Med Sci Sports Exerc. 1991 Mar;23(3):334-42. PMID: 2020272.**
[7] **Black J, Chesher GB, Starmer GA, Egger G. The painlessness of the long distance runner. Med J Aust. 1979 Jun 2;1(11):522-3. PMID: 470706.**
[8] **Kemppainen P, Pertovaara A, Huopaniemi T, Johansson G, Karonen SL. Modification of dental pain and cutaneous thermal sensitivity by physical exercise in man. Brain Res. 1985 Dec 23;360(1-2):33-40. doi: 10.1016/0006-8993(85)91217-x. PMID: 3000534.**
[9] **Kemppainen P, Pertovaara A, Huopaniemi T, Johansson G. Elevation of dental pain threshold induced in man by physical exercise is not reversed by cyproheptadine-mediated suppression of growth hormone release. Neurosci Lett. 1986 Oct 20;70(3):388-92. doi: 10.1016/0304-3940(86)90585-9. PMID: 3774237.**
[10] **Kemppainen P, Paalasmaa P, Pertovaara A, Alila A, Johansson G. Dexamethasone attenuates exercise-induced dental analgesia in man. Brain Res. 1990 Jun 11;519(1-2):329-32. doi: 10.1016/0006-8993(90)90096-t. PMID: 2168784.**

**Comments from the Reviewer 5:**

1. **The purpose of meta-analysis is to increase sample size to answer one question by pooling studies with same design using same intervention and same control.**

**Four of the 5 studies included in this meta-analysis had different study designs. The writer couldn't find the one named "GREWAL et al, 2007" in table 1 in PubMed.**

**The study by Azab et al in 2022 compared clinical Pilates exercise combined with conventional physical therapy to control of conventional physical therapy in poly JIA patients.**

**The study by Sandstedt et al in 2013 compared exercise program consisted of one hundred 2-footed jumps with a rope and standardized muscle strength exercise with control of no exercise in polyarticular or extended oligoarticular arthritis.**

**The study by Tarakci et al in 2012 compared land-based exercised with control of no exercise in JIA patients with strengthening exercise excluded.**

**The study by Takken et al in 2003 compared aquatic fitness with control of no exercise in JIA patients in a phase of remission without medication of no longer than 6 months in the absence of joint pain, tenderness and/or morning stiffness, and an erythrocyte sedimentation rate within normal limits.**

**The results of those studies should be pooled together because interventions and controls were different.**

**Response: Thank you for pointing out this deficiency. The subject of this meta-analysis study is the effect of exercise on juvenile arthritis. The inclusion criteria for the intervention was any type of exercise, i.e., physical activity, which is defined by WHO as “any physical movement produced by skeletal muscle that results in energy expenditure [1].” and physical activity can be categorized into different types depending on the exercise mode, intensity, frequency, and duration. The types of exercises mentioned in this meta-analysis (Pilates exercise, aerobic exercise, resistance exercise, aquatic exercise) are all within the scope of “physical activity”, so in terms of interventions, this study is consistent. In the control group, our inclusion criterion was a non-exercise intervention, i.e., not actively performing any physical activity. Apparently, all included studies also met the criteria. However, there was one study with the presence of traditional physiotherapy in the control group. However, after analysis, this study was not a source of heterogeneity, and we felt that it could also be included in our meta-analysis to increase its completeness. Moreover, in earlier published meta-analyses on exercise interventions [2], studies with physical therapy in the control group were also included. In summary, we believe that the results of the included studies can be meta-analyzed and have a certain degree of reliability.**

**In addition, literature on "GREWAL et al, 2007" that you cannot find on PubMed is available at the following URL: https://pubmed.ncbi.nlm.nih.gov/17907238/**

**Reference:**

[1] **Caspersen CJ, Powell KE, Christenson GM. Physical activity, exercise, and physical fitness: definitions and distinctions for health-related research. Public Health Rep. 1985 Mar-Apr;100(2):126-31. PMID: 3920711; PMCID: PMC1424733.**
[2] **Kuntze G, Nesbitt C, Whittaker JL, Nettel-Aguirre A, Toomey C, Esau S, Doyle-Baker PK, Shank J, Brooks J, Benseler S, Emery CA. Exercise Therapy in Juvenile Idiopathic Arthritis: A Systematic Review and Meta-Analysis. Arch Phys Med Rehabil. 2018 Jan;99(1):178-193.e1. doi: 10.1016/j.apmr.2017.05.030. Epub 2017 Jul 18. PMID: 28729171.**
2. **The data was not accurately documented in the manuscript. In table 1 which was the main results of the study, control group of Takken 2003 was "assessment-only" rather than "community physical therapist", and control group of Tarakci 2013(which was 2012 actually) was "no exercise on waiting list" rather than "waist-list" (which I assume was meant to be wait-list).**

**Response: We apologize for the confusion. Based on your suggestion, we have rechecked the descriptions in the original literature and modified the tables accordingly. (Table 1)**
