# Supplementary material for: Effect of exercise training on heath, quality of life, exercise capacity in juvenile idiopathic arthritis: a meta-analysis of randomized controlled trials
Source: Pediatr Rheumatol Online J. 2024 Mar 4;22:33. doi: 10.1186/s12969-024-00967-3 (PMC10910763; doi:10.1186/s12969-024-00967-3)
Supplement: Supplementary file 5 — Supplementary Material 5 [file 12969_2024_967_MOESM5_ESM.docx]

**Dear editor,**

I am pleased to submit the enclosed manuscript, entitled “**Effect of exercise training on heath, quality of life, exercise capacity in juvenile idiopathic arthritis: a meta-analysis of randomized controlled trials**” as a meta-analysis to ***Pediatric Rheumatology*.**

Juvenile idiopathic arthritis is the most common rheumatic disease in children and adolescents, and current pharmacological treatment has limitations, while exercise is a potential treatment option. However, the effectiveness and safety of exercise for juvenile idiopathic arthritis is unclear at this time. This meta-analysis aimed to investigate the effect of exercise training on quality of life, exercise capacity, and pain in juvenile arthritis. We used traditional meta-analysis methods to draw the following conclusions:

1. **Exercise training does not significantly improve the quality of life and exercise capacity in juvenile idiopathic arthritis.**
2. **Exercise training relieves the pain levels of** **juvenile idiopathic arthritis.**
3. **In clinical practice, exercise training is safe and can be used as an adjunctive treatment for juvenile idiopathic arthritis.**

This meta-analysis provides an evidence-based reference for clinical practitioners to develop exercise prescriptions for the prognosis of chemotherapy patients. We believe that our narrative review may be more helpful to policymakers, service commissioners, and carers. Therefore, we decided to submit our work to ***Pediatric Rheumatology.*** I hope you find this manuscript suitable for publication in your journal. Thank you for your consideration.

Sincerely,

Wen-yu Liu,

Weifang Institute of Technology, No.9888 Yunmenshan South Road, Qingzhou City, Shandong Province, China;

Universiti Malaysia Sarawak, Sarawak, East Malaysian Borneo.

(E-mail: 714599308@qq.com)
